# Associations between occupational trajectories at ages 35–55 and later-life cognition in older adults in Chile

**DOI:** 10.1093/geronb/gbag142

**Published:** 2026-07-16

**Authors:** Magdalena Delaporte

**Affiliations:** Population Studies Center and Department of Sociology, University of Pennsylvania, Philadelphia, Pennsylvania, United States; International Max Planck Research School for Population, Health and Data Science, Max Planck Institute for Demographic Research, Rostock, Germany; (Social Sciences Section)

**Keywords:** Life-course, Global aging, Occupation

## Abstract

**Objectives:**

As global rates of Alzheimer’s Disease and Related Dementias (AD/ADRD) increase, understanding the factors that contribute to cognitive aging is crucial. This study examined the association between occupational skill trajectories between ages 35 and 55 and cognitive function among older Chilean adults aged 60 and above. It explored how different patterns of occupational trajectories relate to cognitive performance.

**Methods:**

The analysis used data from the Chile-Cog study, which applied the Harmonized Cognitive Assessment Protocol (HCAP) to a nationally representative sample of Chilean adults in 2019. These cognitive assessments were linked to detailed occupational histories from the Chilean Social Protection Survey (SPS). Using Sequence Analysis, occupational trajectories between ages 35 and 55 were classified into nine groups for women and six groups for men. Cognitive function was measured using a composite cognitive score based on the HCAP items. Linear regression models estimated the associations between occupational trajectory groups and cognitive outcomes, separately for women (*n *= 1,009) and men (*n *= 769).

**Results:**

Individuals with longer participation in middle- and high-skill occupations showed better cognitive performance at older ages, whereas prolonged periods in low-skill jobs or not being employed were associated with lower scores. However, no strong evidence emerged on associations between specific trajectories of occupational mobility and cognitive outcomes.

**Discussion:**

The findings suggest that cumulative time in higher-skill occupations may be more important for cognitive aging than occupational mobility itself. This study contributes to understanding how occupational trajectories shape cognition in later life and offers insights for countries undergoing economic and demographic transitions.

Population aging is accelerating worldwide, with the proportion of adults aged 65 and above projected to outnumber children under 18 by 2080 (United Nations, 2024). This demographic shift carries several challenges, including a growing prevalence of Alzheimer’s Disease and Related Dementias (AD/ADRD). With the number of people affected by AD/ADRD projected to increase rapidly globally (Sosa-Ortiz et al., 2012), cognitive decline will pose significant challenges to healthcare and pension systems, making it valuable to understand the factors that are associated with healthy cognitive aging. As individuals spend a substantial portion of their lives in employment, occupational experiences may play a crucial role in shaping cognition.

Research has found important links between employment and cognitive function, as labor-force participation in adulthood has been found to predict cognition in later life (Delaporte, 2025; Wahrendorf et al., 2021). In particular, more complex occupations are associated with better cognitive outcomes in older adults (Edwin et al., 2024; Kobayashi & Feldman, 2019; Pool et al., 2016). Additionally, occupational complexity has been associated with a reduced risk of AD/ADRD (Andel et al., 2005; Finkel et al., 2009). The positive associations between high-skill occupations and cognition may persist beyond working life, as evidence suggests that individuals who retire from cognitively demanding jobs tend to maintain better cognitive performance and show slower cognitive decline after retirement (Kajitani et al., 2017).

Gender also plays a key role in how employment is associated with cognitive outcomes, as labor-force patterns often differ substantially between men and women (Mayeda et al., 2020). Some evidence suggests that women may benefit from specific types of work arrangements, such as part-time employment (Bertogg & Leist, 2023).

Although most evidence comes from high-income countries, there is increasing interest in understanding these dynamics in other contexts. Labor markets and employment trajectories in low- and middle-income countries (LMICs) often differ substantially from those in wealthier nations, potentially shaping cognitive outcomes in distinct ways. Nevertheless, emerging findings from LMICs suggest broadly similar patterns (Gutierrez et al., 2024; Rodriguez & Saenz, 2022; Yu et al., 2024).

Most existing studies, however, rely on a single measure of main lifetime occupation or short employment histories, limiting insight regarding more dynamic and longer work experiences. As a result, it is still unclear whether the type of occupational trajectory, such as holding a stable job versus transitioning across jobs with different skill levels, has distinct implications for cognitive aging.

This paper aims to address this gap by examining the relationship between occupational trajectories across midlife and later-life cognitive function among older Chilean adults. Chile offers an interesting case for studying these processes, as its older population benefited from the country’s economic expansion during adulthood, gaining extended employment opportunities and improved health-care access (Núñez et al., 2020; Parro & Reyes, 2019). At the same time, their formative years, which are crucial for shaping employment and health trajectories (Montez & Hayward, 2011), were spent in the context of a middle-income country.

Unlike experiences from other high-income countries, the recent Chilean economic and demographic transitions might reflect a broader global trend characterized by the simultaneous experience of population aging and economic development. By studying older Chilean adults, this research contributes to a broader understanding of how occupational trajectories predict cognitive health in diverse socioeconomic and demographic settings.

## Background: the Chilean context

Between 1980 and 2016, the period covered by the data used in this study, Chile experienced profound economic and structural transformations. During the 1980s, Chile began a transition toward a liberalized, export-oriented economy, following earlier structural adjustments in the 1970s. By the 1990s and early 2000s, Chile had consolidated itself as one of Latin America’s most stable economies, with sustained GDP growth and declining poverty rates (Contreras, 2001). By 2010, Chile had joined the OECD, and in 2012, the World Bank formalized its status as a high-income country.

These macroeconomic shifts were accompanied by substantial changes in the labor market. First, Chile experienced an increase in female labor-force participation (Figure S1). At the same time, high-skill occupations, such as teachers, engineers, and health workers, expanded, while employment in medium- and low-skill occupations declined. Medium-skill occupations include skilled agricultural jobs, secretaries, and sales workers, whereas low-skill occupations are concentrated in manual labor in mining, agriculture, and forestry, as well as domestic service. Consistent with these patterns, the proportion of men employed in agricultural, forestry, and fishery work declined, alongside an increase in professional and technical occupations (Figure S2).

Despite these trends, the Chilean labor market continues to exhibit significant heterogeneity in terms of mobility (Albagli et al., 2023), job quality (Ruiz-Tagle & Sehnbruch, 2015), and gender differentiation (Didier, 2024). Informality remains prevalent (Parro & Reyes, 2019), especially among older workers (Madero-Cabib & Biehl, 2021) and those with lower educational attainment. Many individuals also experience work trajectories that include transitions in and out of the labor force (Madero-Cabib & Cabello-Hutt, 2022). These patterns may be particularly relevant for understanding disparities in later-life cognition (Wahrendorf et al., 2021).

## Methods

### Data

Occupational data come from the Social Protection Survey (SPS), a longitudinal survey representative of Chileans aged 18 and older. To date, SPS has implemented survey waves in 2002, 2004, 2006, 2009, 2016, 2019/2020, and 2024 (Subsecretaría de Previsión Social, 2016), in which information on employment, income, health, and other socioeconomic characteristics has been collected.

In 2019, the Chile-Cog study interviewed a subsample of SPS respondents aged 60 and older (*N *= 2,033) and applied the Harmonized Cognitive Assessment Protocol (HCAP) to assess cognitive function in a nationally representative sample of Chilean older adults. More information on Chile-Cog and its parent survey (SPS) can be found in Delaporte (2025) and Mani et al. (2025).

Earlier SPS survey waves can be merged with the Chile-Cog data, allowing linkages between employment trajectories over decades and cognitive scores for older adults. In each SPS wave, respondents reported their employment history since their last interview. For new respondents in each wave, work histories since 1980 were collected. If a respondent did not participate in a specific wave, they reported their employment history between their last interview and the current one. For this study, I used data from all waves between 2002 and 2016, which include employment data from 1980 to 2016.

For the analysis, I restricted the sample to Chile-Cog respondents who participated in the SPS 2016 wave (*N *= 1,992) to ensure all respondents had employment information through 2016, the most recent SPS wave before Chile-Cog data collection. Moreover, I further restricted the sample to Chile-Cog respondents with at least six years of occupational data available (*N *= 1,947). Although missing values in skill level were imputed as described in the next section, this restriction ensured that there was sufficient occupational history to support reliable imputation.

Finally, I excluded respondents with missing data on cognitive scores. Missing cognitive scores were primarily due to illiteracy (*N *= 144) or very low cognitive scores that prevented the respondent from completing the interview (*N *= 25). After these restrictions, no individuals had missing information on the covariates. The final analytical sample consisted of 1,009 women and 769 men. See Figure S3 for the flowchart of the sample selection criteria and the final number of observations by sex.

### Measures

#### Cognitive function

To measure cognition, I used a total cognitive score that considered all the items assessed in the HCAP battery. The instrument included questions assessing the respondents’ cognitive status in five domains: orientation, memory, executive function, language, and visuospatial.

Moreover, I estimated models with each of the five cognitive domains separately as the dependent variables. I also used the 30-point version of the Mini-Mental State Examination (MMSE), which has been validated and previously used in Chile as a summary measure of cognitive function (Quiroga L et al., 2004). Using these different dimensions of cognition allowed me to explore whether the relationships between occupation and cognitive function vary by domain.

All the cognitive scores were standardized to have a mean of 0 and a standard deviation (*SD*) of 1, facilitating comparisons across various measures. I used data prepared by the Gateway to Global Aging Data, which imputes missing values in cognitive scores when the missingness is due to physical impairment or refusal (Gateway to Global Aging Data, 2023). Because missing values resulting from illiteracy or very low cognitive scores were not imputed, 169 observations were excluded, as noted above.

#### Occupation

Employment histories and occupations were obtained from the SPS. For each SPS wave, occupation is coded using ISCO-88 or ISCO-08 classifications based on International Labour Organization standards (International Labour Organization, 2024). I followed the ISCO classification to assess the skill level associated with each occupational code. These levels are defined as a function of the complexity and the range of tasks and duties performed in an occupation (International Labour Organization, 2024). Table S1 shows the skill level associated with each occupation category: high-, medium-, or low-skill level. I did not include armed forces or other occupations not elsewhere classified.

In addition, I created a separate category to include respondents who were not working at a given age. This group included both individuals actively seeking employment and those out of the labor force. For women, I further disaggregated this group by reasons for inactivity to identify those who were not working due to caregiving responsibilities (e.g., taking care of children or older adults, household chores, or other family obligations).

Respondents reported employment information for different time periods. For example, one person reported being employed from April 2005 to October 2010, while another reported employment from April 2005 to June 2006. To standardize these reports, I restructured the dataset to obtain monthly employment information from January 1980 to December 2015. I then calculated the number of months each person was employed and not employed in each year. If a person was employed for six or more months in a given year, I assigned them the most frequently reported skill level for that year. I applied a similar procedure for non-employment, assigning either “not-employed” or “caregiving” to women based on the most frequently reported status each year if they were not employed for at least six months.

Finally, I calculated each respondent’s age for every year between 1980 and 2015. I then restricted the sequences to ages 35 to 55 to minimize missing data, avoiding cases where respondents were too old to have employment records in the early years or too young to have complete data in the later years. Despite this restriction, some missing occupational data remained, primarily at the beginning of the sequences. For instance, a respondent who was 40 years old in 1980 lacked occupation data for ages 35 through 39. To address this, I restricted the sample to individuals with at least six observed years of data and imputed missing values. Table S2 presents the distribution of missing occupation data among Chile-Cog respondents interviewed in the 2016 SPS wave. It shows that 62.5% of the sample have complete occupational histories, while 35.4% have between one and 15 years of missing data. I limited the analytical sample to those with no more than 15 missing years (*N *= 1,947) to make the imputations more accurate. Table S3 shows the distribution of missing data by age for the final analytical sample (*N *= 1,778), confirming that most gaps occurred at the start of the sequences, particularly for individuals over age 35 in 1980.

To address missing occupational data, I used the *seqimpute* package in R, developed by Emery et al. (2022). This method fills in gaps by identifying similar employment sequences across the sample, using information from both earlier and later points in each individual’s trajectory. See Supplementary Methods for details on the imputation procedure.

#### Other covariates

As additional covariates, I used age in 2019 and educational attainment. Education was coded following the ISCED international classifications and considered completed at one of the following levels: less than primary education, primary education, lower-secondary education, upper-secondary education, or any college or equivalent. I also included the highest parental education to capture early childhood socioeconomic conditions. Parental education was coded as 1 if at least one of the parents attended school. If one was missing, I used the educational level of the parent with valid data. If information on both parents was missing, I coded it as missing. Moreover, I controlled for marital status in 2016, which can be associated with cognition in later life (Liu et al., 2020).

### Empirical strategy

For the estimations, I followed two steps to examine how occupational trajectories are associated with cognitive function in later life.

#### Occupation

First, I used sequence analysis to identify patterns in occupational trajectories between ages 35 and 55, separately for women and men. Each respondent’s occupational history was represented as a sequence in which each age had an employment state: low-skilled, medium-skilled, high-skilled, caregiver, or not-employed for women and low-skilled, medium-skilled, high-skilled, or not-employed for men. Sequence analysis allowed the classification of these occupational histories, capturing the pathways individuals follow across the life course.

In order to group the individual sequences into meaningful categories, I used Lesnard’s Dynamic Hamming Distance matrix to calculate the distance between the sequences of each respondent, as used in previous literature (Kobayashi & Feldman, 2019). After the pairwise distances for each sequence were obtained, I created clusters using Ward’s linkage to minimize within-group variance. To ensure sufficient sample size and interpretability, I subsequently combined clusters that exhibited similar patterns of mobility. For instance, transitions from low- to medium-skill occupations were grouped with transitions from medium- to high-skill occupations. While this approach may obscure some differences in the cognitive demands associated with finer occupational trajectories, the sample sizes posed limitations, and the current approach allowed for sufficient cluster sizes for more reliable estimates. The resulting groups reflected common occupational patterns, such as long-term stability in a particular skill level, transitions across skill levels, or frequent periods of caregiving.

#### Cognition

Second, I assessed how occupational clusters are associated with cognitive outcomes in later life using a linear regression model. I used Ordinary Least Squares to estimate the following equation:


$$C_{i}=\alpha +\sum \beta_{j}O_{ji}+\gamma Z_{i}+\varepsilon_{i},$$

where, $C_{i}$ represents the standardized score for individual i, encompassing the total cognitive score, the MMSE, and the domain-specific scores. The occupation clusters $O_{ji}$ estimated in the first stage were included. $O_{ji}$ equals 1 if respondent i belongs to cluster j. $Z_{i}$ represents the covariates age, educational attainment, parental education, and marital status. All models were estimated using survey weights to account for the Chile-Cog sampling design.

## Results

### Descriptive statistics

Table 1 presents descriptive statistics separately by sex. On average, women scored significantly lower than men on the MMSE. However, no significant sex differences were observed in the total cognitive score or most cognitive domains, with the exception of memory and visuospatial function, as women scored significantly higher on memory, while men outperformed women in visuospatial abilities.

**Table 1:** Summary statistics of the analytic sample by sex.

Variables	Women (*n *= 1,009)		Men (*n *= 769)		*p*
	Mean	%	Mean	%	
**Cognition**					
Total cognitive score	0.0		0.0		.43
MMSE	0.1		0.2		.02
Orientation	0.2		0.1		.81
Memory	0.2		−0.0		.00
Executive function	0.1		0.2		.08
Language	0.1		0.1		.61
Visuospatial	0.0		0.2		.00
**Occupation (number of years)**					
Non-employed	3.4		1.8		.00
Caregiver	7.5		–		
Low-skill	3.4		2.5		.00
Medium-skill	4.7		13.8		.00
High-skill	2.0		2.9		.00
**Covariates**					
Age, in years	70.7		70.5		.59
Completed educational attainment					
Less than primary		25.1		22.4	.18
Primary		31.4		31.2	.93
Lower secondary		16.9		16.6	.87
Upper secondary		17.3		19.1	.34
Any college		9.2		10.7	.31
Parental education					
No education		43.7		40.6	.19
Some education		41.4		40.4	.68
Missing		14.9		19.0	.02
Marital status					
Married/cohabiting		47.1		78.4	.00
Separated/divorced		12.9		7.4	.00
Widowed		23.9		6.5	.00
Never married		16.2		7.7	.00

*Note*. MMSE = Mini-Mental State Examination.

Substantial differences emerged in occupational histories. Women spent, on average, 7.5 years in caregiving roles. They also spent more time unemployed (3.4 vs 1.8  years). Conversely, men accumulated significantly more years in medium-skill and high-skill occupations. Although women spent slightly more time in low-skill jobs, the difference was small.

There were no significant sex differences in age, respondent, or parental education. However, marital status varied significantly by sex. While 78.4% of men were married or cohabiting, this was true for only 47.1% of women. A higher proportion of women were widowed or had never been married, and women were also more likely to be separated or divorced.

### Occupational sequences

#### Women

**Figure 1:**
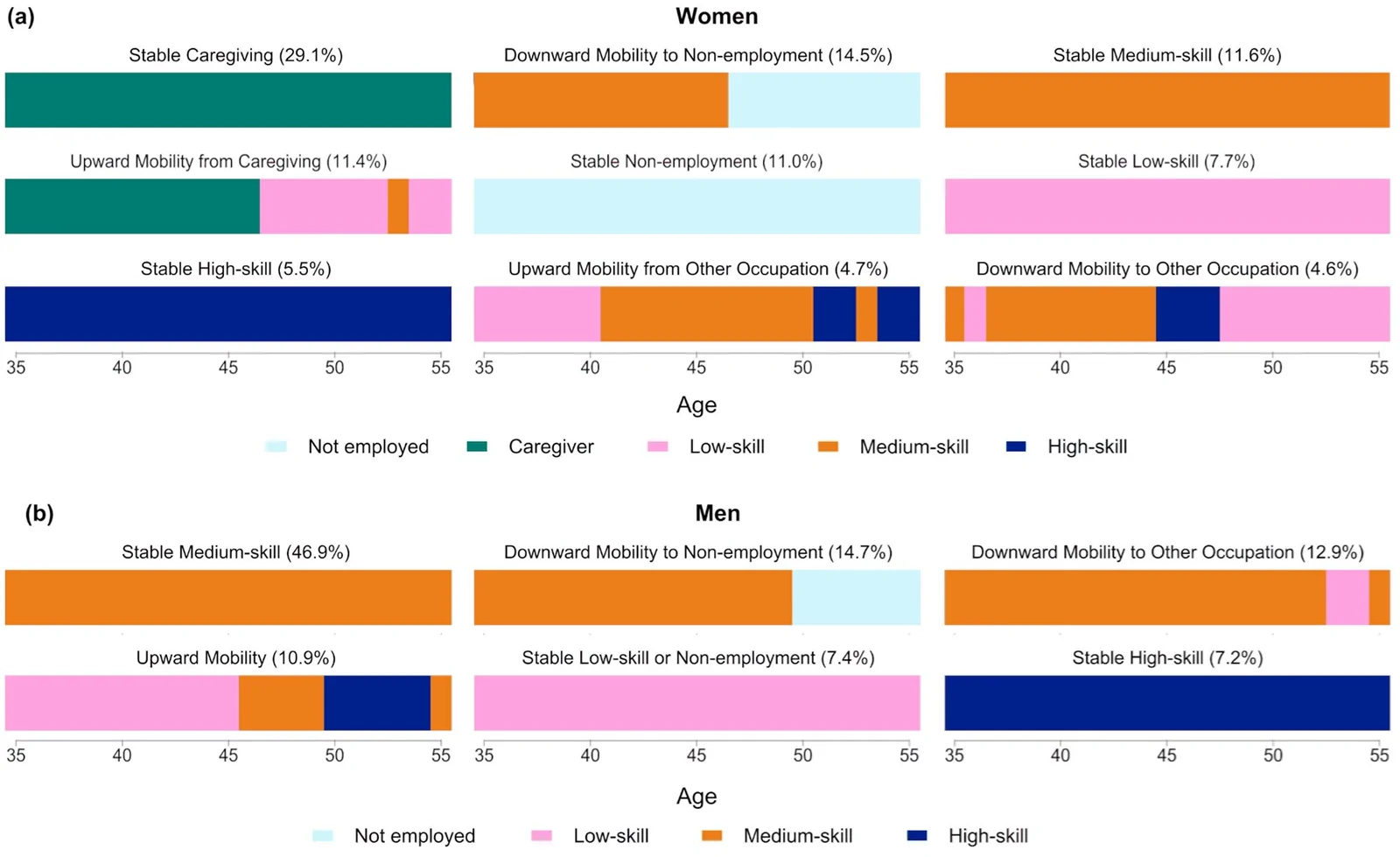
Prototypical occupational clusters based on sequence analysis.

Sequence analysis revealed nine distinct clusters of employment trajectories for women, as shown in Panel (a) in Figure 1, which are ordered from the most-frequent to the least-frequent group. The largest cluster, Stable Caregiving (29.1% of women), was characterized by sustained absence from the labor force due to caregiving responsibilities between ages 35 and 55. The second-largest cluster, Downward Mobility to Non-employment (14.5%), consisted of women who engaged in the labor force but exited during mid-adulthood. The Stable Medium-skill cluster (11.6%) was characterized by women who followed a stable pattern. The following three clusters showed upward mobility patterns characterized by entering the labor force after caregiving responsibilities (11.4%), non-employment (11.0%), and low-skill occupations (7.7%). Next was the Stable High-skill cluster, in which 5.5% of women spent their adulthood in high-skill occupations. This group was followed by the Upward-mobility from Other Occupation cluster (4.7%), characterized by women in the labor force who moved from lower-skill occupations to higher-skill occupations between the ages 35 and 55. Lastly, a small percentage of the sample (4.6%) followed a Downward-mobility pattern.

Table S4 shows descriptive characteristics across occupational trajectory clusters among women. Among women, occupational trajectory clusters had sharp contrasts in education, family background, and marital status. The Stable Caregiving group, the largest cluster, was among the oldest and most disadvantaged, with low individual and parental education and a high proportion of widows. At the opposite extreme, the Stable High-skill group, though small in size, was the most educationally advantaged, with nearly all women completing higher education and reporting high levels of parental schooling, but with comparatively lower rates of marriage or cohabitation. Across groups, patterns of marital status mirrored educational and occupational stratification: women in more advantaged clusters were less likely to be married, whereas widowhood was especially prevalent in downward-mobility groups. Overall, these findings highlighted the intersecting roles of education, family background, and marital histories in shaping women’s occupational pathways.

#### Men

Panel (b) in Figure 1 shows six distinct clusters resulting from sequence analysis. The most common group was Stable Medium-skill, comprising 46.9% of men. The Downward Mobility to Non-employment cluster followed, with 14.7%, characterized by men who exited the labor force around age 50 (before the legal retirement age, i.e., 65 for men). Next, 12.9% of the men followed a Downward Mobility to Other Occupations trajectory. The three remaining clusters were Upward Mobility (10.9%), Stable Low-skill or Non-employment (7.4%), and Stable High-skill (7.2%).

Table S5 presents the descriptive statistics for men categorized by their occupational-trajectory clusters. The Upward Mobility group stood out as the most educationally advantaged, with higher levels of both individual and parental education, as well as the highest proportion of married or cohabiting individuals. At the other end, the Stable Low-skill or Non-employment cluster was characterized by low levels of education across generations and a comparatively high share of never-married individuals. The Stable Medium-skill group fell between these groups, with a concentration of individuals who completed only primary schooling, limited tertiary education, and relatively similar levels of parental education to the Stable Low-skill group, but with a higher proportion married or cohabiting. In general, the heterogeneity observed within clusters revealed that patterns of education and marital status were closely related to occupational trajectories.

### Associations between occupational clusters and cognition

#### Women

**Figure 2:**
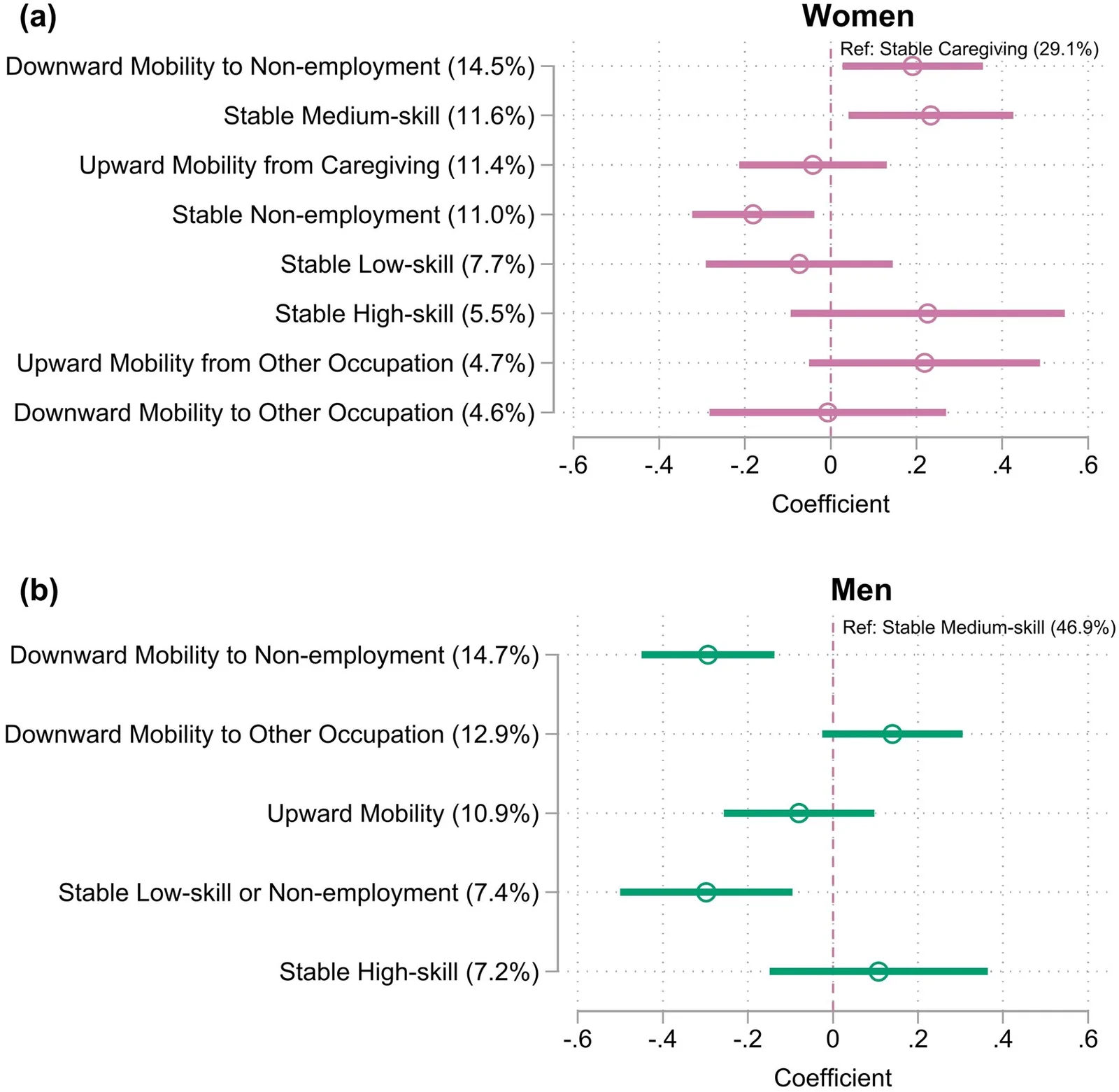
Association between occupation clusters and cognitive function. (i) Models control for age in 2019, completed educational attainment, parental education, and marital status in 2016. (ii) Full tables are available in Supplementary Tables 6 and 10 for women and men, respectively. (iii) Confidence intervals at a 95% level.

Figure 2 shows that, among women, only a few occupational trajectories were significantly associated with cognitive outcomes in later life compared to those in the Stable Caregiving cluster. Women in the Stable Medium-skill group scored about 0.25 *SD* points higher on the standardized cognitive test than women in the caregiving reference category. Women who followed a trajectory of Downward Mobility to non-employment showed a smaller but still significant cognitive advantage, scoring nearly 0.20 *SD* points above the reference group. In contrast, women with Stable Non-employment patterns had lower cognitive scores compared to those in the Stable Caregiving cluster. Other occupational patterns, including Upward Mobility from Caregiving, Stable Low-skill, and other clusters, were not significantly different from the reference caregiving group. However, although not statistically significant, women in the Stable High-skill and Upward Mobility trajectories had higher cognitive scores compared to the reference category. Table S7 reports pairwise comparisons across occupational clusters obtained by re-estimating the models using alternative reference groups, allowing direct comparisons between any two clusters. Results for women indicated that the Stable Non-employment group showed consistently lower cognitive function relative to most other occupational trajectories.

I also examined heterogeneities by age group (60–69 years and 70+ years). Results, available in Supplementary Material, suggest some differences across groups, with stronger associations among women aged 70+.

As a robustness check, I estimated an additional model that controlled for employment status in 2017. The associations between occupational clusters and cognition were largely unchanged (Table S9).

#### Men

Panel (b) in Figure 2 shows that, compared to those in the Stable Medium-skill cluster, men who experienced Downward Mobility to Non-employment had notably lower cognitive scores: 0.29 *SD* points below the reference group. Additionally, men in the Stable Low-skill or Non-employment cluster also scored significantly lower, with cognitive scores 0.30 *SD* points below those in a Stable Medium-skill trajectory. Other occupational trajectories did not show statistically significant differences in cognitive function relative to the Stable Medium-skill group. Table S11 reports pairwise comparisons across clusters, indicating consistently lower cognitive function among men in the Downward Mobility to Non-employment group. Heterogeneities by age group are also available (Table S12). Results were robust to controlling for employment status (Table S13).

### Additional analysis

#### Number of years in each occupation

Considering the potential influence of the occupational composition within each cluster on the results shown in Figure 2, I estimated additional models using the number of years spent in each occupational state as an independent variable. For example, the positive and significant association observed for women in the Downward Mobility to Non-employment cluster may have been driven by the fact that many of these women had accumulated a substantial number of years in medium- or high-skill occupations before transitioning out of the labor force.

**Figure 3:**
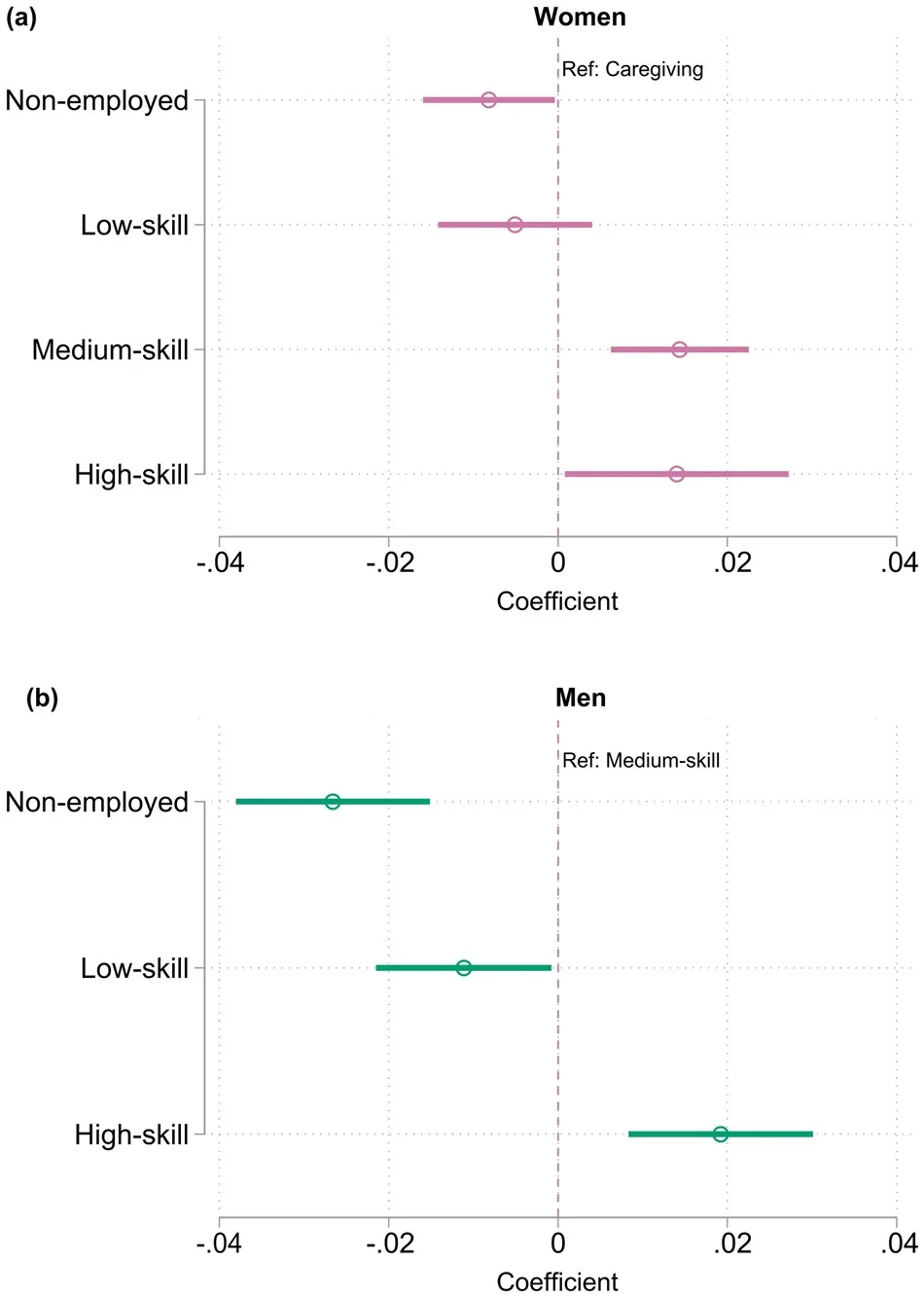
Associations between the number of years in each occupation and cognitive function. (i) Models control for age in 2019, completed educational attainment, parental education, and marital status in 2016. (ii) Full tables are available in Supplementary Tables 14 and 18 for women and men, respectively. (iii) Confidence intervals at a 95% level.

Figure 3 shows the result from these regressions (full tables are available in the Supplementary Material). Panel (a) shows that an additional year spent not employed was significantly associated with lower cognitive scores in later life compared to an additional year of caregiving. In contrast, an additional year spent in medium-skill and high-skill occupations was positively associated with total cognitive scores. The coefficient for low-skill occupations was negative but not statistically significant.

For men, results in Panel (b) indicated that spending more years in non-employment was associated with significantly lower cognitive scores compared to years in a medium-skill occupation. Similarly, time spent in low-skill occupations was also negatively associated with cognition, although the coefficient was smaller compared to those not employed. In contrast, more years spent in high-skill occupations were positively and significantly associated with better cognitive function.

#### Other cognitive domains

I used the MMSE and the five cognitive domains as outcomes to examine how occupational trajectories were associated with different cognitive measures. Results indicate that the associations between occupation and cognition may be driven primarily by executive function, as other cognitive domains showed no consistent link with skill level (see Supplementary Material).

### Sensitivity analysis

Given that the results described previously include imputed skill levels when occupation is missing, I ran a series of sensitivity analyses to ensure that the results were robust to different imputations (see Supplementary Material for details).

As shown in Table S22, the proportions of respondents in each cluster were similar regardless of the imputation procedure. However, when using the original dataset without imputations and coding the missingness as an additional category, a higher proportion of respondents was observed in more stable clusters. Given that missing cases were concentrated at earlier ages, the missingness might obscure some mobility patterns occurring between ages 35 and 45, leading to a higher proportion of respondents in stable trajectories.

Regression results (Supplementary Tables 23–26) indicated that associations between employment trajectories and cognition were robust to different imputation methods, as well as to the original non-imputed dataset.

## Discussion

By assessing the relationship between mid-life occupational trajectories and cognition in later life, this study extends prior work by adopting a life-course perspective and identifying distinct occupational skill trajectories and transitions. This approach is based on the importance of cumulative occupational experiences rather than occupational status at a single point in time.

Results show that employment trajectories differ between women and men, which highlights the gendered patterns of labor-force engagement. A substantial proportion of women in the sample remained outside the labor force throughout adulthood, while most men spent most of their adulthood in medium-skill occupations.

The associations between occupational trajectories and cognition were weak. Among women, while some downward mobility or stable medium-skill employment patterns were associated with better cognitive performance, most other trajectories, including stable low-skill histories, did not differ significantly from the caregiving reference group. Similarly, for men, only two clusters, those characterized by downward mobility or persistent low-skill/non-employment, showed significant cognitive disadvantages relative to the Stable Medium-skill group. These results align with previous literature. In particular, Kobayashi and Feldman (2019) find a weak association between occupation and cognition when examining clustered-occupational trajectories.

An interesting finding is that analyses using the number of years spent in each occupational category reveal a clear gradient: more years spent not employed were associated with lower cognitive scores, while additional years in high-skill occupations were linked to higher scores. These findings suggest that the type of labor-force engagement, whether stable or more mixed, may be less important than the cumulative time spent in higher-skill roles.

The findings of this study contribute to the growing body of literature on the relationship between occupational trajectories and cognitive function in later life. Consistent with prior research, the results suggest that sustained engagement in the labor force is linked to better cognitive performance for older adults in Chile (Delaporte, 2025). In addition, cognitively complex occupations are associated with higher cognition (Andel et al., 2005; Kajitani et al., 2017; Kobayashi & Feldman, 2019; Pool et al., 2016). Additional analysis indicates that the association between occupational skills and cognition might be mediated mainly by executive function. These findings align with the cognitive reserve hypothesis, which posits that mentally stimulating activities, including complex occupational tasks, can build resilience against cognitive decline (Stern, 2002).

Additionally, the observed associations between employment patterns and cognition in Chile are consistent with studies from other countries, including Mexico and South Africa, where employment in high-status occupations or sustained work participation has been linked to better cognitive outcomes (Gutierrez et al., 2024; Rodriguez & Saenz, 2022; Yu et al., 2024). These similarities suggest that employment-related cognitive benefits may extend across diverse economic and policy contexts.

Sex differences emerged as a critical dimension in the occupation and cognition associations. Consistent with prior studies, women’s employment histories were distinct from those of men, with many women experiencing long periods of non-employment. Moreover, results suggest that, for women, an additional year in a low-skill occupation was not different from an additional year out of the labor force due to caregiving responsibilities. This suggests that the skill requirements and tasks of caregiving might be comparable to those needed in low-skill occupations. Few studies have addressed this, but results indicate that older caregivers have better cognition than their peers (Bertrand et al., 2012; Elayoubi et al., 2023; Su, 2023). As research from high-income countries suggests, the association between labor-force engagement and cognition differs by gender (Mayeda et al., 2020). This raises important questions about how labor-force policies and care responsibilities shape women’s cognitive trajectories differently from men’s.

Although evidence suggests that there is a link between occupation and cognition, employment may influence cognition through different pathways, including social interaction, physical activity, and exposure to occupational stressors or environmental hazards. Workplaces provide opportunities for social interactions that have been linked to cognitive benefits (Ihle et al., 2017; Stine-Morrow & Manavbasi, 2022), while physically demanding jobs may play a part in cognitive decline (Zotcheva et al., 2023). Conversely, unstable or high-stress occupations have been associated with worse cognitive function (Burgard & Lin, 2013), as have jobs involving exposure to substances such as pesticides and pollution (Baldi et al., 2011; Hayden et al., 2010; Starks et al., 2012; Thompson et al., 2022). Future research should further disentangle these mechanisms and their interactions in different occupational contexts.

It is important to mention that this study is not without limitations. First, it is important to acknowledge the potential role of mortality selection. As there is evidence of mortality differentials by educational level in Chile (Sandoval et al., 2022), the analytical sample likely reflects more educated, healthier individuals. Hence, the findings may underrepresent individuals with less favorable occupational trajectories or worse cognition, limiting the generalizability of the results to the broader Chilean population. Second, the occupational data rely on retrospective self-reports, which may be subject to recall bias, particularly when reported for several years in the past. This can lead to measurement errors, especially for respondents with multiple employment disruptions. As a result, occupational trajectories may appear smoother than they actually were, as mobility patterns were less accurately captured, particularly before 2002. Third, sample size constraints limit the level of disaggregation in some clusters, which may obscure differences in the cognitive demands associated with occupational trajectories. Fourth, occupational trajectories might also be affected by health and cognition, making it difficult to ascertain the direction of the relation. Lastly, classifications on skill levels do not capture other relevant aspects of jobs, such as stress, cognitive and physical demands, or autonomy, all of which also may affect cognition.

Despite these limitations, this study makes a valuable contribution by using detailed life-course employment data and sequence analysis to understand how work histories are associated with cognitive aging in a rapidly aging population. In addition, insights from Chile may help anticipate cognitive-aging patterns in contexts characterized by simultaneous population aging and economic development.

## Conclusion

In conclusion, this study shows that occupational complexity is associated with cognitive function in older Chilean adults. In particular, the findings suggest that the duration spent in occupations with higher cognitive demands may be more important than the specific occupational trajectories. These results reinforce the protective role of cognitively demanding work. Policies aimed at promoting labor-force engagement for women, reducing employment precarity, and ensuring safe and stimulating work environments could play a crucial role in supporting cognitive health across the life course.

## Supplementary Material

gbag142_Supplementary_Data

## Data Availability

The data used in this study are publicly available. The Social Protection Survey data can be accessed through the Undersecretary of Social Security at https://www.previsionsocial.gob.cl. The Chile-Cog data, including the imputed cognitive scores, are available through the Gateway to Global Aging Data at https://g2aging.org. This study was not preregistered.
