# Supplementary material for: Associations between occupational trajectories at ages 35–55 and later-life cognition in older adults in Chile
Source: J Gerontol B Psychol Sci Soc Sci. 2026 Jul 16;81(10):gbag142. doi: 10.1093/geronb/gbag142 (PMC13544970; doi:10.1093/geronb/gbag142)

The Journals of Gerontology, Series B: Psychological Sciences and Social Sciences
Supplementary Material: Delaporte. Associations Between Occupational Trajectories at Ages 35-55 and Later-Life Cognition in Older Adults in Chile.

Supplementary Methods

Imputation

To address missing occupation data, I used the seqimpute package in R. This method fills in gaps by identifying similar employment sequences across the sample, using information from both earlier and later points in each individual's trajectory. The imputation model also incorporated gender and education to improve accuracy. For each missing observation, the algorithm generated five plausible occupational skill levels. I assigned the most frequent value among the five imputations. For example, if the imputed values were medium, medium, high, high, and medium, the final assignment for that case was medium-skill. In cases without a clear mode, such as medium, medium, high, high, and low, I assigned the highest skill level of the most frequent values (in this example, high-skill). For robustness, I compared results using an alternative approach in which the lowest frequent value was selected instead. The findings were consistent across both methods. Additional details and comparisons are provided in the Supplementary Table 22.

Additionally, I ran a series of sensitivity analysis to ensure that the results are robust to imputation. Supplementary Table 22 shows the distribution of respondents in each occupational cluster using two sets of imputations and the original data, with missing values coded as an additional state. As mentioned above, Imputation 1 (or mode-max) consisted of choosing the most frequent value from five imputations, for each respondent and age with missing occupation. In the case in which no mode was available, the highest most repeated value among those five was chosen. Imputation 2, on the other side, chose the lowest most repeated value among the five imputations in case there is no mode. Column (3) shows the distribution by cluster when using the missing value as an additional state, instead of dropping respondents with missing values.

Supplementary Tables

Supplementary Table 1

Skill levels according to the ILO classifications

**Broad skill level**	**ISCO-08**	**ISCO-88**
High skill	1. Managers	1. Legislators, senior officials, and managers
	2. Professionals	2. Professionals
	3. Technicians and associate professionals	3. Technicians and associated professionals
Medium skill	4. Clerical support workers	4. Clerks
	5. Service and sales workers	5. Service workers and shop and market sales workers
	6. Skilled agricultural, forestry, and fishery workers	6. Skilled agricultural and fishery workers
	7. Craft and related trade workers	7. Craft and related trade workers
	8. Plant and machine operators, and assemblers	8. Plant and machine operators, and assemblers
Low skill	9. Elementary occupations	9. Elementary occupations
Armed forces	0. Armed forces occupations	0. Armed forces
Not elsewhere classified	X. Not elsewhere classified	X. Not elsewhere classified

Supplementary Table 2

Distribution of number of years with missing occupation data for Chile-Cog respondents interviewed in SPS 2016

Number of years with missing data	Obs.	%
0	1,245	62.5
1	128	6.4
2	95	4.8
3	64	3.2
4	76	3.8
5	42	2.1
6	44	2.2
7	52	2.6
8	41	2.1
9	28	1.4
10	21	1.1
11	32	1.6
12	23	1.2
13	24	1.2
14	24	1.2
15	8	0.4
16	12	0.6
17	8	0.4
18	5	0.3
19	6	0.3
20	4	0.2
21	10	0.5
Total	1,992	100.0

Supplementary Table 3

Proportion of missing information on occupation at each age

Age	Missing cases		Valid cases	
	Obs.	%	Obs.	%
35	234	26.3	1,544	73.7
36	206	23.0	1,572	77.0
37	187	20.9	1,591	79.1
38	169	18.9	1,609	81.1
39	143	15.9	1,636	84.1
40	123	13.7	1,655	86.3
41	103	11.4	1,676	88.6
42	85	9.4	1,694	90.6
43	71	7.9	1,707	92.1
44	63	7.0	1,716	93.0
45	58	6.5	1,720	93.5
46	41	4.6	1,737	95.4
47	36	4.0	1,743	96.0
48	26	3.0	1,752	97.0
49	20	2.3	1,759	97.7
50	14	1.6	1,765	98.4
51	12	1.3	1,767	98.7
52	13	1.4	1,766	98.6
53	13	1.4	1,765	98.6
54	10	1.1	1,768	98.9
55	11	1.3	1,767	98.7

Supplementary Table 4

Summary statistics by cluster, women

	%
**Stable Caregiving (N=294, 29.1%)**	
Age	70.0
Educational attainment	
Less than primary	27.4
Primary	35.1
Lower secondary	18.2
Upper secondary	15.3
Any college	4.1
Parental education	
No education	52.1
Some education	30.3
Missing	17.6
Marital status	
Married/cohabiting	63.3
Separared/divorced	7.9
Widowed	24.2
Never married	4.5
**Downward Mobility to Non-employment (N=146, 14.5%)**	
Age	68.2
Educational attainment	
Less than primary	18.5
Primary	23.9
Lower secondary	21.7
Upper secondary	29.7
Any college	6.2
Parental education	
No education	46.4
Some education	39.0
Missing	14.5
Marital status	
Married/cohabiting	52.3
Separared/divorced	10.1
Widowed	26.2
Never married	11.4
**Stable Medium-skill (N=117, 11.6%)**	
Age	68.5
Educational attainment	
Less than primary	12.0
Primary	35.3
Lower secondary	19.7
Upper secondary	28.5
Any college	4.5

Continued on next page

Supplementary Table 4

Summary statistics by cluster, women (continued)

	%
Parental education	
No education	25.4
Some education	66.9
Missing	7.8
Marital status	
Married/cohabiting	31.2
Separared/divorced	18.3
Widowed	24.0
Never married	26.5
**Upward Mobility from Caregiving (N=115, 11.4%)**	
Age	66.2
Educational attainment	
Less than primary	21.4
Primary	43.6
Lower secondary	21.0
Upper secondary	7.0
Any college	7.1
Parental education	
No education	50.4
Some education	35.3
Missing	14.4
Marital status	
Married/cohabiting	59.0
Separared/divorced	22.8
Widowed	13.9
Never married	4.3
**Stable Non-employment (N=111, 11.0%)**	
Age	68.4
Educational attainment	
Less than primary	23.2
Primary	39.2
Lower secondary	9.2
Upper secondary	23.1
Any college	5.2
Parental education	
No education	44.3
Some education	37.2
Missing	18.5
Marital status	
Married/cohabiting	50.3
Separared/divorced	12.0
Widowed	23.8

Continued on next page

Supplementary Table 4

Summary statistics by cluster, women (continued)

	%
Never married	13.9
**Stable Low-skill (N=78, 7.7%)**	
Age	72.8
Educational attainment	
Less than primary	46.5
Primary	35.6
Lower secondary	9.1
Upper secondary	7.2
Any college	1.6
Parental education	
No education	56.7
Some education	33.9
Missing	9.4
Marital status	
Married/cohabiting	36.6
Separared/divorced	17.8
Widowed	13.9
Never married	31.7
**Stable High-skill (N=55, 5.5%)**	
Age	68.5
Educational attainment	
Less than primary	0.0
Primary	0.0
Lower secondary	5.0
Upper secondary	11.3
Any college	83.7
Parental education	
No education	20.1
Some education	79.9
Missing	0.0
Marital status	
Married/cohabiting	41.2
Separared/divorced	16.4
Widowed	12.9
Never married	29.5
**Upward Mobility from Other Occupation (N=47, 4.7%)**	
Age	65.1
Educational attainment	
Less than primary	21.0
Primary	15.3
Lower secondary	18.2
Upper secondary	34.2
Any college	11.4

Continued on next page

Supplementary Table 4

Summary statistics by cluster, women (continued)

	%
Parental education	
No education	39.9
Some education	52.2
Missing	7.9
Marital status	
Married/cohabiting	37.0
Separared/divorced	18.8
Widowed	17.2
Never married	27.0
**Downward Mobility to Other Occupation (N=46, 4.6%)**	
Age	67.5
Educational attainment	
Less than primary	13.7
Primary	31.7
Lower secondary	10.6
Upper secondary	30.0
Any college	14.0
Parental education	
No education	26.1
Some education	63.6
Missing	10.4
Marital status	
Married/cohabiting	36.4
Separared/divorced	23.3
Widowed	26.3
Never married	14.0

Supplementary Table 5

Summary statistics by cluster, men

	%
**Stable Medium-skill (N=361, 46.9%)**	
Age	69.3
Educational attainment	
Less than primary	23.2
Primary	36.1
Lower secondary	11.8
Upper secondary	23.3
Any college	5.7
Parental education	
No education	42.0
Some education	36.3
Missing	21.6
Marital status	
Married/cohabiting	76.7
Separared/divorced	9.5
Widowed	4.2
Never married	9.6
**Downward Mobility to Non-employment (N=113, 14.7%)**	
Age	66.9
Educational attainment	
Less than primary	24.7
Primary	40.0
Lower secondary	17.3
Upper secondary	13.2
Any college	4.8
Parental education	
No education	41.8
Some education	43.9
Missing	14.3
Marital status	
Married/cohabiting	66.3
Separared/divorced	14.1
Widowed	12.8
Never married	6.8
**Downward Mobility to Other Occupation (N=99, 12.9%)**	
Age	66.4
Educational attainment	
Less than primary	12.6
Primary	26.3
Lower secondary	22.2
Upper secondary	19.6
Any college	19.3

Continued on next page

Supplementary Table 5

Summary statistics by cluster, men (continued)

	%
Parental education	
No education	30.7
Some education	51.5
Missing	17.7
Marital status	
Married/cohabiting	87.8
Separared/divorced	5.1
Widowed	2.1
Never married	5.1
**Upward Mobility (N=84, 10.9%)**	
Age	66.3
Educational attainment	
Less than primary	18.7
Primary	23.3
Lower secondary	16.6
Upper secondary	31.8
Any college	9.6
Parental education	
No education	32.9
Some education	57.1
Missing	10.0
Marital status	
Married/cohabiting	76.7
Separared/divorced	4.6
Widowed	4.4
Never married	14.2
**Stable Low-skill or Non-employment (N=57, 7.4%)**	
Age	70.5
Educational attainment	
Less than primary	32.5
Primary	54.1
Lower secondary	0.8
Upper secondary	10.9
Any college	1.6
Parental education	
No education	50.2
Some education	30.0
Missing	19.8
Marital status	
Married/cohabiting	67.5
Separared/divorced	0.6
Widowed	1.8

Continued on next page

Supplementary Table 5

Summary statistics by cluster, men (continued)

	%
Never married	30.0
**Stable High-skill (N=55, 7.2%)**	
Age	68.5
Educational attainment	
Less than primary	1.9
Primary	3.0
Lower secondary	14.8
Upper secondary	18.8
Any college	61.4
Parental education	
No education	17.0
Some education	74.1
Missing	8.8
Marital status	
Married/cohabiting	80.1
Separared/divorced	17.0
Widowed	0.9
Never married	2.0

Supplementary Table 6

Association between occupation clusters and cognitive function, women

	(1)	(2)	(3)	(4)	(5)	(6)	(7)
	MMSE	Total score	Orientation	Memory	Executive function	Language	Visuospatial
**Occupation clusters (Ref: Stable Caregiving)**							
Downward Mobility to Non-employment	0.07	0.19*	0.12	0.14	0.17*	0.17*	0.13
	(0.07)	(0.08)	(0.07)	(0.09)	(0.08)	(0.09)	(0.09)
Stable Medium-skill	0.04	0.23*	0.05	0.20	0.21*	0.23*	-0.06
	(0.08)	(0.10)	(0.08)	(0.11)	(0.09)	(0.10)	(0.11)
Upward Mobility from Caregiving	-0.10	-0.04	-0.07	0.02	-0.05	-0.09	-0.18
	(0.08)	(0.09)	(0.07)	(0.10)	(0.08)	(0.09)	(0.09)
Stable Non-employment	-0.18**	-0.18*	-0.13*	-0.20*	-0.13	-0.07	-0.15
	(0.06)	(0.07)	(0.06)	(0.08)	(0.07)	(0.08)	(0.08)
Stable Low-skill	-0.08	-0.07	0.00	-0.19	0.02	-0.02	0.03
	(0.10)	(0.11)	(0.09)	(0.13)	(0.10)	(0.12)	(0.12)
Stable High-skill	0.16	0.23	0.05	0.17	0.24	-0.05	0.31
	(0.14)	(0.16)	(0.13)	(0.18)	(0.15)	(0.17)	(0.18)
Upward Mobility from Other Occupation	0.24*	0.22	0.01	0.14	0.22	0.13	0.41**
	(0.12)	(0.14)	(0.11)	(0.16)	(0.13)	(0.14)	(0.15)
Downward Mobility to Other Occupation	-0.01	-0.01	-0.09	0.13	-0.13	0.02	0.17
	(0.12)	(0.14)	(0.11)	(0.16)	(0.13)	(0.15)	(0.15)
**Covariates**							
Age	-0.02***	-0.05***	-0.01***	-0.04***	-0.05***	-0.03***	-0.02***
	(0.00)	(0.00)	(0.00)	(0.00)	(0.00)	(0.00)	(0.00)
Educational attainment							
Primary	0.27***	0.45***	0.27***	0.40***	0.39***	0.26***	0.39***
	(0.06)	(0.07)	(0.05)	(0.07)	(0.06)	(0.07)	(0.07)
Lower secondary	0.42***	0.59***	0.24***	0.36***	0.64***	0.24**	0.70***
	(0.07)	(0.08)	(0.06)	(0.09)	(0.08)	(0.09)	(0.09)
Upper secondary	0.38***	0.73***	0.23***	0.51***	0.77***	0.26**	0.73***
	(0.07)	(0.08)	(0.06)	(0.09)	(0.08)	(0.08)	(0.09)
Any college	0.59***	1.27***	0.30**	0.89***	1.26***	0.87***	1.20***
	(0.10)	(0.12)	(0.09)	(0.14)	(0.11)	(0.13)	(0.13)
Parental education							
Some education	0.14**	0.21***	0.10*	0.17**	0.20***	0.18**	0.04
	(0.05)	(0.06)	(0.04)	(0.06)	(0.05)	(0.06)	(0.06)
Missing	-0.01	-0.06	0.07	0.01	-0.11	-0.05	-0.06
	(0.06)	(0.07)	(0.06)	(0.08)	(0.07)	(0.08)	(0.08)
Marital status							
Separated/divorced	-0.02	0.16*	-0.08	0.10	0.19**	0.12	-0.01
	(0.07)	(0.08)	(0.06)	(0.09)	(0.07)	(0.08)	(0.08)
Widowed	-0.08	0.02	-0.13**	-0.02	0.07	-0.03	-0.11
	(0.05)	(0.06)	(0.05)	(0.07)	(0.06)	(0.07)	(0.07)
Never married	-0.15*	-0.03	-0.16*	0.01	-0.03	-0.06	-0.11
	(0.07)	(0.08)	(0.06)	(0.09)	(0.08)	(0.09)	(0.09)
Constant	1.20***	3.00***	1.00***	2.28***	3.14***	1.95***	1.06***
	(0.24)	(0.28)	(0.22)	(0.32)	(0.27)	(0.30)	(0.31)
Observations	1,009	1,009	1,009	1,009	1,009	1,009	1,009

Notes: (i) Reference categories are: less than primary education, parents without formal education and married. (ii) Standard errors on parenthesis. ***, ** and * indicate statistical significance at the 99.9%, 99.0% and 95.0%, respectively.

Supplementary Table 7

Association between occupation clusters (different reference categories) and cognitive function, women

	(1)	(2)	(3)	(4)	(5)	(6)	(7)	(8)	(9)
**Occupation clusters**									
Stable Caregiving	ref.	-0.19*	-0.23*	0.04	0.18*	0.07	-0.23	-0.22	0.01
		(0.08)	(0.10)	(0.09)	(0.07)	(0.11)	(0.16)	(0.14)	(0.14)
Downward Mobility to Non-employment	0.19*	ref.	-0.04	0.23*	0.37***	0.26*	-0.04	-0.03	0.20
	(0.08)		(0.12)	(0.11)	(0.10)	(0.13)	(0.17)	(0.15)	(0.15)
Stable Medium-skill	0.23*	0.04	ref.	0.27*	0.41***	0.31*	0.01	0.01	0.24
	(0.10)	(0.12)		(0.12)	(0.11)	(0.14)	(0.18)	(0.16)	(0.16)
Upward Mobility from Caregiving	-0.04	-0.23*	-0.27*	ref.	0.14	0.03	-0.27	-0.26	-0.03
	(0.09)	(0.11)	(0.12)		(0.10)	(0.13)	(0.18)	(0.15)	(0.16)
Stable Non-employment	-0.18*	-0.37***	-0.41***	-0.14	ref.	-0.11	-0.41*	-0.40**	-0.17
	(0.07)	(0.10)	(0.11)	(0.10)		(0.12)	(0.17)	(0.14)	(0.15)
Stable Low-skill	-0.07	-0.26*	-0.31*	-0.03	0.11	ref.	-0.30	-0.29	-0.07
	(0.11)	(0.13)	(0.14)	(0.13)	(0.12)		(0.19)	(0.17)	(0.17)
Stable High-skill	0.23	0.04	-0.01	0.27	0.41*	0.30	ref.	0.01	0.23
	(0.16)	(0.17)	(0.18)	(0.18)	(0.17)	(0.19)		(0.20)	(0.20)
Upward Mobility from Other Occupation	0.22	0.03	-0.01	0.26	0.40**	0.29	-0.01	ref.	0.23
	(0.14)	(0.15)	(0.16)	(0.15)	(0.14)	(0.17)	(0.20)		(0.19)
Downward Mobility to Other Occupation	-0.01	-0.20	-0.24	0.03	0.17	0.07	-0.23	-0.23	ref.
	(0.14)	(0.15)	(0.16)	(0.16)	(0.15)	(0.17)	(0.20)	(0.19)	

Notes: (i) Models control for age, education, parental education and marital status. (ii) Standard errors on parenthesis. ***, ** and * indicate statistical significance at the 99.9%, 99.0% and 95.0%, respectively.

Supplementary Table 8

Association between occupation clusters and cognitive function by age group, women

	(1) All	(2) Ages 60-69	(3) Ages 70+
**Occupation clusters (Ref: Stable Caregiving)**			
Downward Mobility to Non-employment	0.19*	0.18	0.24
	(0.08)	(0.12)	(0.12)
Stable Medium-skill	0.23*	0.25	0.28
	(0.10)	(0.14)	(0.14)
Upward Mobility from Caregiving	-0.04	-0.04	0.01
	(0.09)	(0.12)	(0.15)
Stable Non-employment	-0.18*	-0.13	-0.30**
	(0.07)	(0.10)	(0.10)
Stable Low-skill	-0.07	0.04	-0.17
	(0.11)	(0.21)	(0.12)
Stable High-skill	0.23	0.20	0.30
	(0.16)	(0.24)	(0.21)
Upward Mobility from Other Occupation	0.22	0.24	0.16
	(0.14)	(0.17)	(0.27)
Downward Mobility to Other Occupation	-0.01	0.21	-0.34
	(0.14)	(0.20)	(0.21)
**Covariates**			
Age	-0.05***	-0.03**	-0.06***
	(0.00)	(0.01)	(0.01)
Educational attainment			
Primary	0.45***	0.75***	0.21**
	(0.07)	(0.11)	(0.08)
Lower secondary	0.59***	0.92***	0.29*
	(0.08)	(0.12)	(0.11)
Upper secondary	0.73***	1.04***	0.43***
	(0.08)	(0.12)	(0.12)
Any college	1.27***	1.48***	1.20***
	(0.12)	(0.18)	(0.17)
Parental education			
Some education	0.21***	0.10	0.32***
	(0.06)	(0.08)	(0.08)
Missing	-0.06	0.05	-0.19*
	(0.07)	(0.11)	(0.09)
Marital status			
Separated/divorced	0.16*	0.16	0.16
	(0.08)	(0.11)	(0.11)
Widowed	0.02	0.10	-0.02
	(0.06)	(0.10)	(0.08)
Never married	-0.03	-0.04	0.04
	(0.08)	(0.12)	(0.11)
Constant	3.00***	1.75*	4.26***
	(0.28)	(0.81)	(0.53)
Observations	1,009	506	503

Notes: (i) Models control for age, education, parental education and marital status. (ii) Standard errors on parenthesis. ***, ** and * indicate statistical significance at the 99.9%, 99.0% and 95.0%, respectively.

Supplementary Table 9

Association between occupation clusters and cognitive function controlling by employment status in 2017, women

	(1)	(2)
**Occupation clusters (Ref: Stable Caregiving)**		
Downward Mobility to Non-employment	0.19*	0.20*
	(0.08)	(0.08)
Stable Medium-skill	0.23*	0.17
	(0.10)	(0.10)
Upward Mobility from Caregiving	-0.04	-0.08
	(0.09)	(0.09)
Stable Non-employment	-0.18*	-0.18*
	(0.07)	(0.07)
Stable Low-skill	-0.07	-0.11
	(0.11)	(0.11)
Stable High-skill	0.23	0.18
	(0.16)	(0.16)
Upward Mobility from Other Occupation	0.22	0.15
	(0.14)	(0.14)
Downward Mobility to Other Occupation	-0.01	-0.03
	(0.14)	(0.14)
**Covariates**		
Age	-0.05***	-0.05***
	(0.00)	(0.00)
Educational attainment		
Primary	0.45***	0.44***
	(0.07)	(0.07)
Lower secondary	0.59***	0.60***
	(0.08)	(0.08)
Upper secondary	0.73***	0.73***
	(0.08)	(0.08)
Any college	1.27***	1.26***
	(0.12)	(0.12)
Parental education		
Some education	0.21***	0.21***
	(0.06)	(0.06)
Missing	-0.06	-0.05
	(0.07)	(0.07)
Marital status		
Separated/divorced	0.16*	0.16*
	(0.08)	(0.08)
Widowed	0.02	0.02
	(0.06)	(0.06)
Never married	-0.03	-0.04
	(0.08)	(0.08)
Employment status		
Working	0.15**	(0.06)
Constant	3.00***	2.83***
	(0.28)	(0.29)
Observations	1,009	1,009

Notes: (i) Reference categories are: less than primary education, parents without formal education, married and not working. (ii) Standard errors on parenthesis. ***, ** and * indicate statistical significance at the 99.9%, 99.0% and 95.0%, respectively.

Supplementary Table 10

Association between occupation clusters and cognitive function, men

	(1) MMSE	(2) Total score	(3) Orientation	(4) Memory	(5) Executive function	(6) Language	(7) Visuospatial
**Occupation clusters (Ref: Stable Medium-skill)**							
Downward Mobility to Non-employment	-0.30***	-0.29***	-0.28***	-0.28**	-0.26***	-0.02	-0.12
	(0.07)	(0.08)	(0.07)	(0.09)	(0.08)	(0.09)	(0.09)
Downward Mobility to Other Occupation	-0.08	0.14	-0.02	0.13	0.11	0.13	0.11
	(0.08)	(0.08)	(0.07)	(0.10)	(0.08)	(0.09)	(0.10)
Upward Mobility	-0.13	-0.08	-0.08	-0.08	-0.04	-0.20*	0.06
	(0.08)	(0.09)	(0.08)	(0.11)	(0.09)	(0.10)	(0.11)
Stable Low-skill or Non-employment	-0.09	-0.30**	-0.05	-0.22	-0.32**	-0.18	0.02
	(0.09)	(0.10)	(0.09)	(0.12)	(0.10)	(0.11)	(0.12)
Stable High-skill	-0.10	0.11	-0.17	0.07	0.13	0.01	0.12
	(0.12)	(0.13)	(0.12)	(0.15)	(0.12)	(0.14)	(0.15)
**Covariates**							
Age	-0.02***	-0.05***	-0.02***	-0.03***	-0.05***	-0.03***	-0.02***
	(0.00)	(0.00)	(0.00)	(0.01)	(0.00)	(0.00)	(0.01)
Educational attainment							
Primary	0.40***	0.43***	0.20**	0.21*	0.50***	0.27**	0.53***
	(0.07)	(0.07)	(0.07)	(0.09)	(0.07)	(0.08)	(0.09)
Lower secondary	0.59***	0.81***	0.36***	0.56***	0.82***	0.45***	0.89***
	(0.09)	(0.10)	(0.09)	(0.11)	(0.09)	(0.11)	(0.11)
Upper secondary	0.53***	0.95***	0.22**	0.61***	1.01***	0.55***	1.00***
	(0.08)	(0.09)	(0.08)	(0.10)	(0.08)	(0.09)	(0.10)
Any college	0.89***	1.34***	0.43***	0.89***	1.37***	0.94***	1.07***
	(0.10)	(0.11)	(0.10)	(0.13)	(0.11)	(0.13)	(0.13)
Parental education							
Some education	0.14*	0.25***	0.07	0.12	0.30***	0.12	0.25***
	(0.06)	(0.06)	(0.05)	(0.07)	(0.06)	(0.07)	(0.07)
Missing	-0.09	-0.02	-0.13	-0.11	0.05	-0.08	0.15
	(0.07)	(0.08)	(0.07)	(0.09)	(0.07)	(0.08)	(0.09)
Marital status							
Separated/divorced	0.13	0.04	0.04	-0.14	0.09	0.42***	0.23*
	(0.09)	(0.10)	(0.09)	(0.12)	(0.09)	(0.11)	(0.11)
Widowed	-0.29*	-0.32*	-0.28*	-0.16	-0.37**	-0.16	-0.10
	(0.12)	(0.13)	(0.11)	(0.15)	(0.12)	(0.14)	(0.15)
Never married	-0.22**	-0.42***	-0.06	-0.28**	-0.46***	-0.17	-0.35***
	(0.08)	(0.09)	(0.08)	(0.11)	(0.09)	(0.10)	(0.11)
Constant	1.26***	2.62***	1.40***	1.90***	2.73***	2.16***	0.95*
	(0.29)	(0.32)	(0.28)	(0.38)	(0.30)	(0.35)	(0.37)
Observations	769	769	769	769	769	769	769

Notes: (i) Reference categories are: less than primary education, parents without formal education and married. (ii) Standard errors on parenthesis. ***, ** and * indicate statistical significance at the 99.9%, 99.0% and 95.0%, respectively.

Supplementary Table 11

Association between occupation clusters (different reference categories) and cognitive function, men

	(1)	(2)	(3)	(4)	(5)	(6)
**Occupation clusters**						
Stable Medium-skill	ref.	0.29***	-0.14	0.08	0.30**	-0.11
		(0.08)	(0.08)	(0.09)	(0.10)	(0.13)
Downward Mobility to Non-employment	-0.29***	ref.	-0.43***	-0.21*	0.00	-0.40**
	(0.08)		(0.10)	(0.11)	(0.12)	(0.14)
Downward Mobility to Other Occupation	0.14	0.43***	ref.	0.22*	0.44***	0.03
	(0.08)	(0.10)		(0.11)	(0.12)	(0.14)
Upward Mobility	-0.08	0.21*	-0.22*	ref.	0.22	-0.19
	(0.09)	(0.11)	(0.11)		(0.13)	(0.15)
Stable Low-skill or Non-employment	-0.30**	-0.00	-0.44***	-0.22	ref.	-0.41*
	(0.10)	(0.12)	(0.12)	(0.13)		(0.16)
Stable High-skill	0.11	0.40**	-0.03	0.19	0.41*	ref.
	(0.13)	(0.14)	(0.14)	(0.15)	(0.16)	

Notes: (i) Models control for age, education, parental education and marital status. (ii) Standard errors on parenthesis. ***, ** and * indicate statistical significance at the 99.9%, 99.0% and 95.0%, respectively.

Supplementary Table 12

Association between occupation clusters and cognitive function by age group, men

	(1)	(2)	(3)
	All	Ages 60-69	Ages 70+
**Occupation clusters (Ref: Stable Medium-skill)**			
Downward Mobility to Non-employment	-0.29***	-0.43***	0.03
	(0.08)	(0.11)	(0.11)
Downward Mobility to Other Occupation	0.14	0.10	0.04
	(0.08)	(0.11)	(0.15)
Upward Mobility	-0.08	-0.05	-0.36*
	(0.09)	(0.12)	(0.15)
Stable Low-skill or Non-employment	-0.30**	-0.41*	-0.15
	(0.10)	(0.17)	(0.12)
Stable High-skill	0.11	-0.01	0.38*
	(0.13)	(0.18)	(0.18)
**Covariates**			
Age	-0.05***	-0.05***	-0.03**
	(0.00)	(0.01)	(0.01)
Educational attainment			
Primary	0.43***	0.60***	0.34***
	(0.07)	(0.12)	(0.09)
Lower secondary	0.81***	0.85***	0.83***
	(0.10)	(0.13)	(0.14)
Upper secondary	0.95***	0.99***	0.98***
	(0.09)	(0.12)	(0.12)
Any college	1.34***	1.54***	0.90***
	(0.11)	(0.16)	(0.16)
Parental education			
Some education	0.25***	0.28**	0.20*
	(0.06)	(0.09)	(0.09)
Missing	-0.02	0.07	-0.11
	(0.08)	(0.12)	(0.10)
Marital status			
Separated/divorced	0.04	0.04	0.02
	(0.10)	(0.13)	(0.16)
Widowed	-0.32*	-1.05***	0.03
	(0.13)	(0.25)	(0.13)
Never married	-0.42***	-0.47***	-0.27*
	(0.09)	(0.13)	(0.13)
Constant	2.62***	3.03***	1.29
	(0.32)	(0.78)	(0.69)
Observations	769	385	384

Notes: (i) Models control for age, education, parental education and marital status. (ii) Standard errors on parenthesis. ***, ** and * indicate statistical significance at the 99.9%, 99.0% and 95.0%, respectively.

Supplementary Table 13

Association between occupation clusters and cognitive function controlling by employment status in 2017, men

	(1)	(2)
**Occupation clusters (Ref: Stable Medium-skill)**		
Downward Mobility to Non-employment	-0.29***	-0.18*
	(0.08)	(0.08)
Downward Mobility to Other Occupation	0.14	0.17*
	(0.08)	(0.08)
Upward Mobility	-0.08	-0.09
	(0.09)	(0.09
Stable Low-skill or Non-employment	-0.30**	-0.25*
	(0.10)	(0.10)
Stable High-skill	0.11	0.12
	(0.13)	(0.13)
**Covariates**		
Age	-0.05***	-0.04***
Educational attainment	(0.00)	(0.00)
Primary	0.43***	0.44***
	(0.07)	(0.07)
Lower secondary	0.81***	0.78***
	(0.10)	(0.10)
Upper secondary	0.95***	0.94***
	(0.09)	(0.08)
Any college	1.34***	1.33***
Parental education	(0.11)	(0.11)
Some education	0.25***	0.25***
	(0.06)	(0.06)
Missing	-0.02	-0.03
Marital status	(0.08)	(0.08)
Separated/divorced	0.04	0.06
	(0.10)	(0.10)
Widowed	-0.32*	-0.34**
	(0.13)	(0.13)
Never married	-0.42***	-0.40***
	(0.09)	(0.09)
Employment status		
Working		0.22***
		(0.06)
Constant	2.62***	2.11***
	(0.32)	(0.35)
Observations	769	769

Notes: (i) Reference categories are: less than primary education, parents without formal education, married and not working. (ii) Standard errors on parenthesis. ***, ** and * indicate statistical significance at the 99.9%, 99.0% and 95.0%, respectively.

Supplementary Table 14

Association between number of years in each occupation and cognitive function, women

	(1)	(2)	(3)	(4)	(5)	(6)	(7)
		**Total**			**Executive**		
	**MMSE**	**score**	**Orientation**	**Memory**	**function**	**Language**	**Visuospatial**
**Occupation (Ref: Caregiving)**							
Non-employed	-0.01*	-0.01*	-0.01*	-0.01	-0.01*	-0.00	-0.01
	(0.00)	(0.00)	(0.00)	(0.00)	(0.00)	(0.00)	(0.00)
Low-skill	-0.01	-0.01	-0.00	-0.01	-0.00	-0.00	0.00
	(0.00)	(0.00)	(0.00)	(0.01)	(0.00)	(0.00)	(0.01)
Medium-skill	0.01	0.01***	0.00	0.01*	0.01***	0.01*	0.00
	(0.00)	(0.00)	(0.00)	(0.00)	(0.00)	(0.00)	(0.00)
High-skill	0.01	0.01*	0.00	0.01	0.01	0.00	0.01
	(0.01)	(0.01)	(0.01)	(0.01)	(0.01)	(0.01)	(0.01)
**Covariates**							
Age	-0.02***	-0.05***	-0.01***	-0.04***	-0.05***	-0.03***	-0.02***
	(0.00)	(0.00)	(0.00)	(0.00)	(0.00)	(0.00)	(0.00)
Educational attainment							
Primary	0.26***	0.43***	0.26***	0.39***	0.37***	0.25***	0.37***
	(0.06)	(0.07)	(0.05)	(0.07)	(0.06)	(0.07)	(0.07)
Lower secondary	0.42***	0.59***	0.25***	0.37***	0.63***	0.24**	0.70***
	(0.07)	(0.08)	(0.06)	(0.09)	(0.08)	(0.09)	(0.09)
Upper secondary	0.36***	0.71***	0.22***	0.49***	0.74***	0.26**	0.74***
	(0.07)	(0.08)	(0.06)	(0.09)	(0.08)	(0.08)	(0.09)
Any college	0.58***	1.21***	0.28**	0.82***	1.21***	0.85***	1.19***
	(0.11)	(0.12)	(0.10)	(0.14)	(0.12)	(0.13)	(0.13)
Parental education							
Some education	0.14**	0.20***	0.09*	0.16**	0.18***	0.19**	0.03
	(0.05)	(0.05)	(0.04)	(0.06)	(0.05)	(0.06)	(0.06)
Missing	-0.01	-0.06	0.07	0.01	-0.10	-0.05	-0.07
	(0.06)	(0.07)	(0.06)	(0.08)	(0.07)	(0.08)	(0.08)
Marital status							
Separated/divorced	-0.03	0.14	-0.09	0.09	0.16*	0.12	-0.03
	(0.07)	(0.08)	(0.06)	(0.09)	(0.07)	(0.08)	(0.08)
Widowed	-0.08	0.01	-0.14**	-0.03	0.05	-0.02	-0.11
	(0.06)	(0.06)	(0.05)	(0.07)	(0.06)	(0.07)	(0.07)
Never married	-0.14*	-0.04	-0.16*	-0.01	-0.03	-0.05	-0.09
	(0.07)	(0.08)	(0.06)	(0.09)	(0.08)	(0.08)	(0.09)
Constant	1.20***	2.97***	0.96***	2.28***	3.09***	1.93***	1.06***
	(0.24)	(0.28)	(0.22)	(0.32)	(0.26)	(0.29)	(0.30)
Observations	1,009	1,009	1,009	1,009	1,009	1,009	1,009

Notes: (i) Reference categories are: less than primary education, parents without formal education and married. (ii) Standard errors on parenthesis. ***, ** and * indicate statistical significance at the 99.9%, 99.0% and 95.0%, respectively.

Supplementary Table 15

Association between number of years in each occupation (different reference categories) and cognitive function, women

	(1)	(2)	(3)	(4)	(5)
**Occupation**					
Caregiver	ref.	0.01*	0.01	-0.01***	-0.01*
		(0.00)	(0.00)	(0.00)	(0.01)
Non-employed	-0.01*	ref.	-0.00	-0.02***	-0.02**
	(0.00)		(0.01)	(0.00)	(0.01)
Low-skill	-0.01	0.00	ref.	-0.02***	-0.02*
	(0.00)	(0.01)		(0.01)	(0.01)
Medium-skill	0.01***	0.02***	0.02***	ref.	0.00
	(0.00)	(0.00)	(0.01)		(0.01)
High-skill	0.01*	0.02**	0.02*	-0.00	ref.
	(0.01)	(0.01)	(0.01)	(0.01)	

Notes: (i) Models control for age, education, parental education and marital status. (ii) Standard errors on parenthesis. ***, ** and * indicate statistical significance at the 99.9%, 99.0% and 95.0%, respectively.

Supplementary Table 16

Association between number of years in each occupation and cognitive function by age group, women

	(1)	(2)	(3)
	All	Ages 60-69	Ages 70+
**Occupation (Ref: Caregiving)**			
Non-employed	-0.01*	-0.00	-0.01**
	(0.00)	(0.01)	(0.00)
Low-skill	-0.01	0.00	-0.01*
	(0.00)	(0.01)	(0.01)
Medium-skill	0.01***	0.01*	0.02*
	(0.00)	(0.01)	(0.01)
High-skill	0.01*	0.02	0.01
	(0.01)	(0.01)	(0.01)
**Covariates**			
Age	-0.05***	-0.03**	-0.06***
	(0.00)	(0.01)	(0.01)
Educational attainment			
Primary	0.43***	0.73***	0.20*
	(0.07)	(0.10)	(0.08)
Lower secondary	0.59***	0.92***	0.26*
	(0.08)	(0.12)	(0.11)
Upper secondary	0.71***	1.02***	0.42***
	(0.08)	(0.12)	(0.12)
Any college	1.21***	1.38***	1.17***
	(0.12)	(0.18)	(0.17)
Parental education			
Some education	0.20***	0.10	0.31***
	(0.05)	(0.08)	(0.08)
Missing	-0.06	0.06	-0.18
	(0.07)	(0.11)	(0.09)
Marital status			
Separated/divorced	0.14	0.13	0.16
	(0.08)	(0.11)	(0.11)
Widowed	0.01	0.09	-0.03
	(0.06)	(0.10)	(0.08)
Never married	-0.04	-0.05	0.03
	(0.08)	(0.12)	(0.11)
Constant	2.97***	1.73*	4.24***
	(0.28)	(0.80)	(0.52)
Observations	1,009	506	503

Notes: (i) Models control for age, education, parental education and marital status. (ii) Standard errors on parenthesis. ***, ** and * indicate statistical significance at the 99.9%, 99.0% and 95.0%, respectively.

Supplementary Table 17

Association between number of years in each occupation and cognitive function controlling by employment status in 2017, women

	(1)	(2)
**Occupation (Ref: Caregiving)**		
Non-employed	-0.01*	-0.01
	(0.00)	(0.00)
Low-skill	-0.01	-0.01
	(0.00)	(0.00)
Medium-skill	0.01***	0.01**
	(0.00)	(0.00)
High-skill	0.01*	0.01
	(0.01)	(0.01)
**Covariates**	-0.05***	-0.05***
Age	(0.00)	(0.00)
Educational attainment		
Primary	0.43***	0.43***
	(0.07)	(0.06)
Lower secondary	0.59***	0.60***
	(0.08)	(0.08)
Upper secondary	0.71***	0.71***
	(0.08)	(0.08)
Any college	1.21***	1.20***
	(0.12)	(0.12)
Parental education		
Some education	0.20***	0.20***
	(0.05)	(0.05)
Missing	-0.06	-0.05
	(0.07)	(0.07)
Marital status		
Separated/divorced	0.14	0.14
	(0.08)	(0.08)
Widowed	0.01	0.01
	(0.06)	(0.06)
Never married	-0.04	-0.05
	(0.08)	(0.08)
Employment status		
Working		0.12*
		(0.06)
Constant	2.97***	2.83***
	(0.28)	(0.29)
Observations	1,009	1,009

Notes: (i) Reference categories are: less than primary education, parents without formal education, married and not working. (ii) Standard errors on parenthesis. ***, ** and * indicate statistical significance at the 99.9%, 99.0% and 95.0%, respectively.

Supplementary Table 18

Association between number of years in each occupation and cognitive function, men

	(1)	(2)	(3)	(4)	(5)	(6)	(7)
	**MMSE**	**Total score**	**Orientation**	**Memory**	**Executive function**	**Language**	**Visuospatial**
**Occupation (Ref: Medium-skill)**							
Non-employed	-0.02***	-0.03***	-0.01**	-0.02**	-0.03***	-0.01	-0.02**
	(0.01)	(0.01)	(0.01)	(0.01)	(0.01)	(0.01)	(0.01)
Low-skill	-0.00	-0.01*	-0.00	-0.01	-0.01*	-0.02**	0.01
	(0.00)	(0.01)	(0.00)	(0.01)	(0.00)	(0.01)	(0.01)
High-skill	-0.00	0.02***	-0.01	0.01*	0.02***	0.01	0.01
	(0.01)	(0.01)	(0.00)	(0.01)	(0.01)	(0.01)	(0.01)
**Covariates**							
Age	-0.02***	-0.05***	-0.02***	-0.03***	-0.05***	-0.04***	-0.02***
	(0.00)	(0.00)	(0.00)	(0.01)	(0.00)	(0.00)	(0.00)
Educational attainment							
Primary	0.39***	0.41***	0.20**	0.19*	0.47***	0.25**	0.51***
	(0.07)	(0.07)	(0.07)	(0.09)	(0.07)	(0.08)	(0.09)
Lower secondary	0.54***	0.76***	0.32***	0.52***	0.77***	0.42***	0.84***
	(0.09)	(0.10)	(0.09)	(0.11)	(0.09)	(0.11)	(0.11)
Upper secondary	0.52***	0.88***	0.23**	0.56***	0.93***	0.48***	0.97***
	(0.08)	(0.09)	(0.08)	(0.10)	(0.08)	(0.10)	(0.10)
Any college	0.88***	1.13***	0.45***	0.74***	1.15***	0.82***	1.01***
	(0.11)	(0.12)	(0.11)	(0.15)	(0.12)	(0.14)	(0.14)
Parental education							
Some education	0.16**	0.26***	0.09	0.12	0.31***	0.11	0.28***
	(0.06)	(0.06)	(0.06)	(0.07)	(0.06)	(0.07)	(0.07)
Missing	-0.08	-0.00	-0.12	-0.09	0.07	-0.07	0.15
	(0.07)	(0.08)	(0.07)	(0.09)	(0.07)	(0.08)	(0.09)
Marital status							
Separated/divorced	0.11	0.01	0.01	-0.17	0.06	0.42***	0.21
	(0.09)	(0.10)	(0.09)	(0.11)	(0.09)	(0.11)	(0.11)
Widowed	-0.34**	-0.34**	-0.34**	-0.20	-0.38**	-0.16	-0.08
	(0.12)	(0.12)	(0.11)	(0.15)	(0.12)	(0.14)	(0.15)
Never married	-0.15	-0.36***	-0.01	-0.23*	-0.40***	-0.16	-0.28**
	(0.08)	(0.09)	(0.08)	(0.11)	(0.08)	(0.10)	(0.11)
Constant	1.13***	2.64***	1.30***	1.87***	2.76***	2.30***	0.96**
	(0.29)	(0.31)	(0.28)	(0.37)	(0.29)	(0.34)	(0.36)
Observations	769	769	769	769	769	769	769

Notes: (i) Reference categories are: less than primary education, parents without formal education and married. (ii) Standard errors on parenthesis. ***, ** and * indicate statistical significance at the 99.9%, 99.0% and 95.0%, respectively.

Supplementary Table 19

Association between number of years in each occupation (different reference categories) and cognitive function, men

	(1)	(2)	(3)	(4)
**Occupation**				
Non-employed	ref.	-0.02*	-0.03***	-0.05***
		(0.01)	(0.01)	(0.01)
Low-skill	0.02*	ref.	-0.01*	-0.03***
	(0.01)		(0.01)	(0.01)
Medium-skill	0.03***	0.01*	ref.	-0.02***
	(0.01)	(0.01)		(0.01)
High-skill	0.05***	0.03***	0.02***	ref.
	(0.01)	(0.01)	(0.01)	

Notes: (i) Models control for age, education, parental education and marital status. (ii) Standard errors on parenthesis. ***, ** and * indicate statistical significance at the 99.9%, 99.0% and 95.0%, respectively.

Supplementary Table 20

Association between number of years in each occupation and cognitive function by age group, men

	(1) All	(2) Ages 60-69	(3) Ages 70+
**Occupation (Ref: Medium-skill)**			
Non-employed	-0.03***	-0.06***	-0.01
	(0.01)	(0.01)	(0.01)
Low-skill	-0.01*	-0.01	-0.01*
	(0.01)	(0.01)	(0.01)
High-skill	0.02***	0.02*	0.02*
	(0.01)	(0.01)	(0.01)
**Covariates**			
Age	-0.05***	-0.05***	-0.03**
	(0.00)	(0.01)	(0.01)
Educational attainment			
Primary	0.41***	0.51***	0.32***
	(0.07)	(0.12)	(0.09)
Lower secondary	0.76***	0.69***	0.81***
	(0.10)	(0.13)	(0.14)
Upper secondary	0.88***	0.82***	0.94***
	(0.09)	(0.13)	(0.12)
Any college	1.13***	1.18***	0.88***
	(0.12)	(0.18)	(0.16)
Parental education			
Some education	0.26***	0.31***	0.20*
	(0.06)	(0.08)	(0.09)
Missing	-0.00	0.09	-0.12
	(0.08)	(0.11)	(0.10)
Marital status			
Separated/divorced	0.01	0.00	0.01
	(0.10)	(0.12)	(0.16)
Widowed	-0.34**	-1.13***	0.01
	(0.12)	(0.24)	(0.13)
Never married	-0.36***	-0.27*	-0.29*
	(0.09)	(0.13)	(0.13)
Constant	2.64***	3.15***	1.33*
	(0.31)	(0.77)	(0.68)
Observations	769	385	384

Notes: (i) Models control for age, education, parental education and marital status. (ii) Standard errors on parenthesis. ***, ** and * indicate statistical significance at the 99.9%, 99.0% and 95.0%, respectively.

Supplementary Table 21

Association between number of years in each occupation and cognitive function controlling by employment status in 2017, men

	(1)	(2)
**Occupation (Ref: Medium-skill)**		
Non-employed	-0.03***	-0.02**
	(0.01)	(0.01)
Low-skill	-0.01*	-0.01*
	(0.01)	(0.01)
High-skill	0.02***	0.02***
	(0.01)	(0.01)
**Covariates**		
Age	-0.05***	-0.04***
	(0.00)	(0.00)
Educational attainment		
Primary	0.41***	0.41***
	(0.07)	(0.07)
Lower secondary	0.76***	0.73***
	(0.10)	(0.09)
Upper secondary	0.88***	0.86***
	(0.09)	(0.09)
Any college	1.13***	1.11***
	(0.12)	(0.12)
Parental education		
Some education	0.26***	0.24***
	(0.06)	(0.06)
Missing	-0.00	-0.01
	(0.08)	(0.08)
Marital status		
Separated/divorced	0.01	0.04
	(0.10)	(0.10)
Widowed	-0.34**	-0.36**
	(0.12)	(0.12)
Never married	-0.36***	-0.36***
	(0.09)	(0.09)
Employment status		
Working		0.24***
		(0.06)
Constant	2.64***	2.13***
	(0.31)	(0.33)
Observations	769	769

Notes: (i) Reference categories are: less than primary education, parents without formal education, married and not working. (ii) Standard errors on parenthesis. ***, ** and * indicate statistical significance at the 99.9%, 99.0% and 95.0%, respectively.

Supplementary Table 22

Distribution of respondents by cluster based on different imputation results

	(1)<br>Imputation 1<br>Mode-max	(2)<br>Imputation 2<br>Mode-min	(3)<br>Original,<br>no imputation
**Women (N=1,009)**			
Stable non-employment	11.0	8.7	9.5
Stable caregiving	29.1	29.1	33.8
Stable low-skill	7.7	8.1	9.3
Stable medium-skill	11.6	10.7	11.8
Stable high-skill	5.5	5.5	6.5
Downward mobility to non-employment	14.5	15.4	10.8
Downward mobility to other occupation	4.6	5.9	4.3
Upward mobility from caregiving	11.4	12.6	10.1
Upward mobility from other occupation	4.7	4.0	3.9
**Men (N=769)**			
Stable low-skill or non-employment	7.4	7.3	9.0
Stable medium-skill	46.9	45.8	49.9
Stable high-skill	7.2	7.2	8.6
Downward mobility to non-employment	14.7	15.2	10.3
Downward mobility to other occupation	12.9	13.7	10.7
Upward mobility	10.9	10.9	11.6

Notes: (i) Imputation 1 generates five imputations for each case with missing skill level, based on respondent and age. The most frequent (modal) value across the five imputations is selected. If no single mode exists (i.e., there is a tie), the highest skill level among the tied values is chosen. Imputation 2 follows the same procedure, but in the event of a tie, it selects the lowest skill level among the tied values. The original version does not impute missing skill levels; instead, it assigns a new category to missing values to avoid losing observations. (ii) Reference categories are: less than primary education, parents without formal education and married. (iii) Standard errors on parenthesis. ***, ** and * indicate statistical significance at the 99.9%, 99.0% and 95.0%, respectively.

Supplementary Table 23

Association between occupation clusters and total cognitive function using different imputation results, women

	(1) Imputation 1 Mode-max	(2) Imputation 2 Mode-min	(3) Original, no imputation
**Occupation clusters (Ref: Stable Caregiving)**			
Stable Non-employment	-0.18*	-0.18*	-0.12
	(0.07)	(0.08)	(0.08)
Stable Low-skill	-0.07	-0.10	-0.10
	(0.11)	(0.11)	(0.10)
Stable Medium-skill	0.23*	0.24*	0.26**
	(0.10)	(0.10)	(0.10)
Stable High-skill	0.23	0.23	0.26
	(0.16)	(0.16)	(0.15)
Downward Mobility to Non-employment	0.19*	0.19*	0.16
	(0.08)	(0.08)	(0.09)
Downward Mobility to Other Occupation	-0.01	0.04	0.10
	(0.14)	(0.13)	(0.14)
Upward Mobility from Caregiving	-0.04	-0.09	-0.04
	(0.09)	(0.08)	(0.09)
Upward Mobility from Other occupation	0.22	0.26	0.10
	(0.14)	(0.14)	(0.14)
**Covariates**			
Age	-0.05***	-0.05***	-0.05***
	(0.00)	(0.00)	(0.00)
Educational attainment			
Primary	0.45***	0.45***	0.45***
	(0.07)	(0.07)	(0.07)
Lower secondary	0.59***	0.59***	0.60***
	(0.08)	(0.08)	(0.08)
Upper secondary	0.73***	0.73***	0.73***
	(0.08)	(0.08)	(0.08)
Any college	1.27***	1.27***	1.23***
	(0.12)	(0.12)	(0.12)
Parental education			
Some education	0.21***	0.21***	0.20***
	(0.06)	(0.06)	(0.06)
Missing	-0.06	-0.07	-0.08
	(0.07)	(0.07)	(0.07)
Marital status			
Separated/divorced	0.16*	0.17*	0.16*
	(0.08)	(0.08)	(0.08)
Widowed	0.02	0.02	0.02
	(0.06)	(0.06)	(0.06)
Never married	-0.03	-0.04	-0.02
	(0.08)	(0.08)	(0.08)
Constant	3.00***	3.01***	2.91***
	(0.28)	(0.28)	(0.29)
Observations	1,009	1,009	1,009

Notes: (i) Imputation 1 generates five imputations for each case with missing skill level, based on respondent and age. The most frequent (modal) value across the five imputations is selected. If no single mode exists (i.e., there is a tie), the highest skill level among the tied values is chosen. Imputation 2 follows the same procedure, but in the event of a tie, it selects the lowest skill level among the tied values. The original version does not impute missing skill levels; instead, it assigns a new category to missing values to avoid losing observations. (ii) Reference categories are: less than primary education, parents without formal education and married. (iii) Standard errors on parenthesis. ***, ** and * indicate statistical significance at the 99.9%, 99.0% and 95.0%, respectively.

Supplementary Table 24

Association between occupation clusters and total cognitive function using different imputation results, men

	(1)<br>Imputation 1<br>Mode-max	(2)<br>Imputation 2<br>Mode-min	(3)<br>Original,<br>no imputation
**Occupation clusters (Ref: Stable Medium-skill)**			
Stable Low-skill or Non-employment	-0.30**	-0.17	-0.30**
	(0.10)	(0.10)	(0.10)
Stable High-skill	0.11	0.09	0.12
	(0.13)	(0.13)	(0.12)
Downward Mobility to Non-employment	-0.29***	-0.32***	-0.47***
	(0.08)	(0.08)	(0.09)
Downward Mobility to Other Occupation	0.14	0.07	0.12
	(0.08)	(0.08)	(0.09)
Upward Mobility	-0.08	-0.10	0.00
	(0.09)	(0.09)	(0.09)
**Covariates**			
Age	-0.05***	-0.05***	-0.05***
	(0.00)	(0.00)	(0.00)
Educational attainment			
Primary	0.43***	0.44***	0.40***
	(0.07)	(0.07)	(0.07)
Lower secondary	0.81***	0.84***	0.78***
	(0.10)	(0.10)	(0.10)
Upper secondary	0.95***	0.96***	0.94***
	(0.09)	(0.09)	(0.08)
Any college	1.34***	1.36***	1.30***
	(0.11)	(0.11)	(0.11)
Parental education			
Some education	0.25***	0.27***	0.24***
	(0.06)	(0.06)	(0.06)
Missing	-0.02	-0.01	-0.03
	(0.08)	(0.08)	(0.08)
Marital status			
Separated/divorced	0.04	0.05	-0.03
	(0.10)	(0.10)	(0.10)
Widowed	-0.32*	-0.30*	-0.32**
	(0.13)	(0.13)	(0.12)
Never married	-0.42***	-0.45***	-0.44***
	(0.09)	(0.09)	(0.09)
Constant	2.62***	2.66***	2.65***
	(0.32)	(0.32)	(0.33)
Observations	769	769	769

Notes: (i) Imputation 1 generates five imputations for each case with missing skill level, based on respondent and age. The most frequent (modal) value across the five imputations is selected. If no single mode exists (i.e., there is a tie), the highest skill level among the tied values is chosen. Imputation 2 follows the same procedure, but in the event of a tie, it selects the lowest skill level among the tied values. The original version does not impute missing skill levels; instead, it assigns a new category to missing values to avoid losing observations. (ii) Reference categories are: less than primary education, parents without formal education and married. (iii) Standard errors on parenthesis. ***, ** and * indicate statistical significance at the 99.9%, 99.0% and 95.0%, respectively.

Supplementary Table 25

Association between number of years in each occupation and total cognitive function using different imputation results, women

	(1) Imputation 1 Mode-max	(2) Imputation 2 Mode-min	(3) Original, no imputation
**Occupation (Ref: Caregiving)**			
Non-employed	-0.01*	-0.01*	-0.01*
	(0.00)	(0.00)	(0.00)
Low-skill	-0.01	-0.01	-0.00
	(0.00)	(0.00)	(0.01)
Medium-skill	0.01***	0.01***	0.02***
	(0.00)	(0.00)	(0.00)
High-skill	0.01*	0.01*	0.02*
	(0.01)	(0.01)	(0.01)
Missing			-0.02
			(0.01)
**Covariates**			
Age	-0.05***	-0.05***	-0.04***
	(0.00)	(0.00)	(0.01)
Educational attainment			
Primary	0.43***	0.43***	0.44***
	(0.07)	(0.06)	(0.06)
Lower secondary	0.59***	0.59***	0.60***
	(0.08)	(0.08)	(0.08)
Upper secondary	0.71***	0.71***	0.72***
	(0.08)	(0.08)	(0.08)
Any college	1.21***	1.21***	1.20***
	(0.12)	(0.12)	(0.12)
Parental education			
Some education	0.20***	0.20***	0.20***
	(0.05)	(0.05)	(0.05)
Missing	-0.06	-0.06	-0.05
	(0.07)	(0.07)	(0.07)
Marital status			
Separated/divorced	0.14	0.14	0.13
	(0.08)	(0.08)	(0.08)
Widowed	0.01	0.01	0.01
	(0.06)	(0.06)	(0.06)
Never married	-0.04	-0.04	-0.05
	(0.08)	(0.08)	(0.08)
Constant	2.97***	2.95***	2.54***
	(0.28)	(0.28)	(0.36)
Observations	1,009	1,009	1,009

Notes: (i) Imputation 1 generates five imputations for each case with missing skill level, based on respondent and age. The most frequent (modal) value across the five imputations is selected. If no single mode exists (i.e., there is a tie), the highest skill level among the tied values is chosen. Imputation 2 follows the same procedure, but in the event of a tie, it selects the lowest skill level among the tied values. The original version does not impute missing skill levels; instead, it assigns a new category to missing values to avoid losing observations. (ii) Reference categories are: less than primary education, parents without formal education and married. (iii) Standard errors32 on parenthesis. ***, ** and * indicate statistical significance at the 99.9%, 99.0% and 95.0%, respectively.

Supplementary Table 26

Association between number of years in each occupation and total cognitive function using different imputation results, men

	(1) Imputation 1 Mode-max	(2) Imputation 2 Mode-min	(3) Original, no imputation
**Occupation (Ref: Medium-skill)**			
Non-employed	-0.03***	-0.03***	-0.03***
	(0.01)	(0.01)	(0.01)
Low-skill	-0.01*	-0.01*	-0.01
	(0.01)	(0.01)	(0.01)
High-skill	0.02***	0.02***	0.02***
	(0.01)	(0.01)	(0.01)
Missing			0.02
			(0.01)
**Covariates**			
Age	-0.05***	-0.05***	-0.05***
	(0.00)	(0.00)	(0.01)
Educational attainment			
Primary	0.41***	0.41***	0.40***
	(0.07)	(0.07)	(0.07)
Lower secondary	0.76***	0.76***	0.74***
	(0.10)	(0.10)	(0.10)
Upper secondary	0.88***	0.88***	0.87***
	(0.09)	(0.09)	(0.09)
Any college	1.13***	1.13***	1.11***
	(0.12)	(0.12)	(0.12)
Parental education			
Some education	0.26***	0.26***	0.26***
	(0.06)	(0.06)	(0.06)
Missing	-0.00	-0.00	-0.01
	(0.08)	(0.08)	(0.08)
Marital status			
Separated/divorced	0.01	0.01	0.00
	(0.10)	(0.10)	(0.10)
Widowed	-0.34**	-0.34**	-0.34**
	(0.12)	(0.12)	(0.12)
Never married	-0.36***	-0.36***	-0.36***
	(0.09)	(0.09)	(0.09)
Constant	2.64***	2.62***	2.90***
	(0.31)	(0.31)	(0.36)
Observations	769	769	769

Notes: (i) Imputation 1 generates five imputations for each case with missing skill level, based on respondent and age. The most frequent (modal) value across the five imputations is selected. If no single mode exists (i.e., there is a tie), the highest skill level among the tied values is chosen. Imputation 2 follows the same procedure, but in the event of a tie, it selects the lowest skill level among the tied values. The original version does not impute missing skill levels; instead, it assigns a new category to missing values to avoid losing observations. (ii) Reference categories are: less than primary education, parents without formal education and married. (iii) Standard errors on parenthesis. ***, ** and * indicate statistical significance at the 99.9%, 99.0% and 95.0%, respectively.

Figures

Supplementary Figure 1

Trends in occupational skills by gender in Chile, ages 25-64

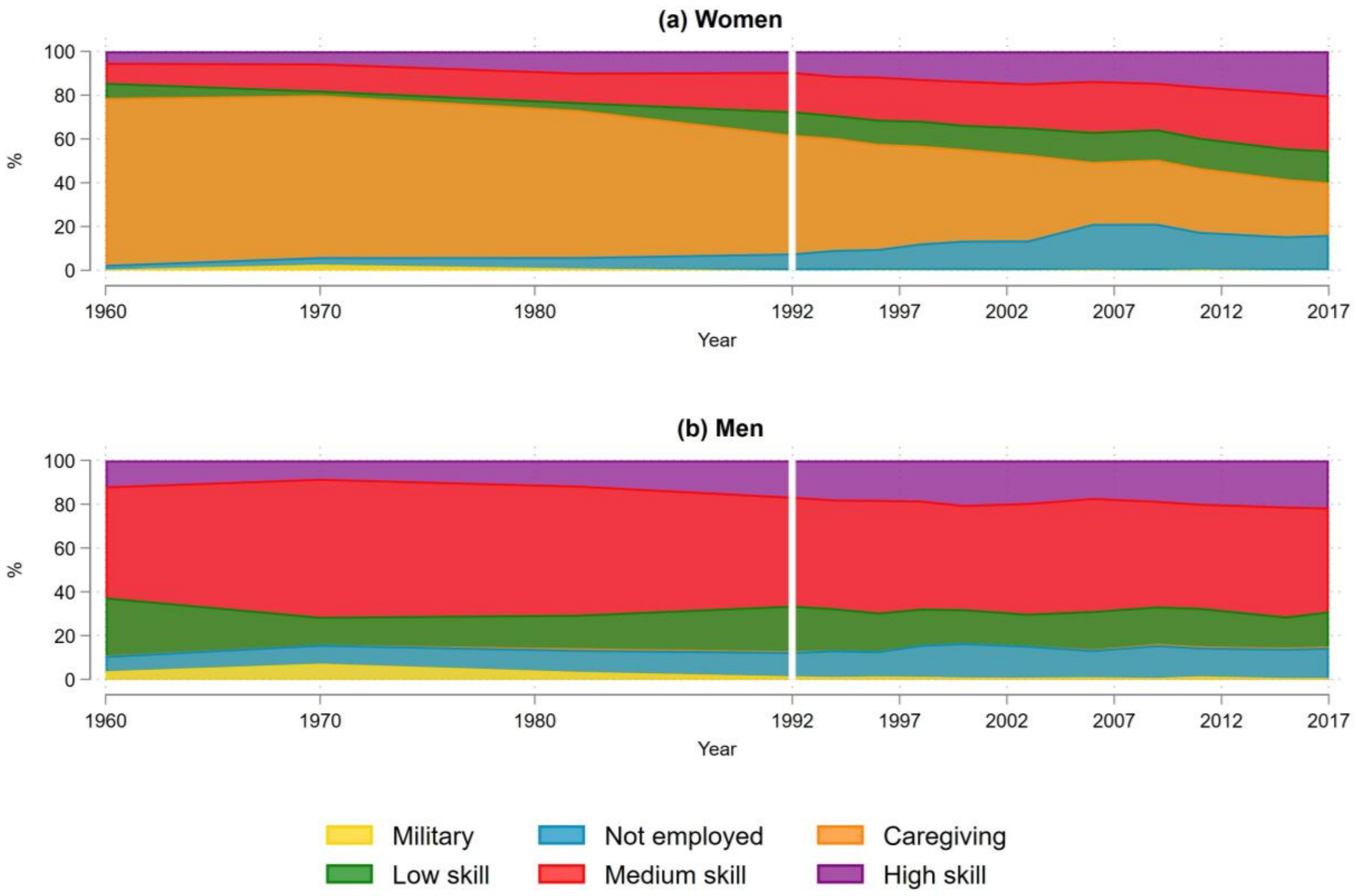

Notes: Data for years 1960, 1970 and 1982 comes from Census data obtained through IPUMS. Data for years 1992, 1994, 1996, 1998, 2000, 2003, 2006, 2009, 2011, 2015 and 2017 comes from the National Socioeconomic Characterization Survey, CASEN.

Supplementary Figure 2

Trends in occupational categories among employed individuals by gender in Chile, ages 25-64

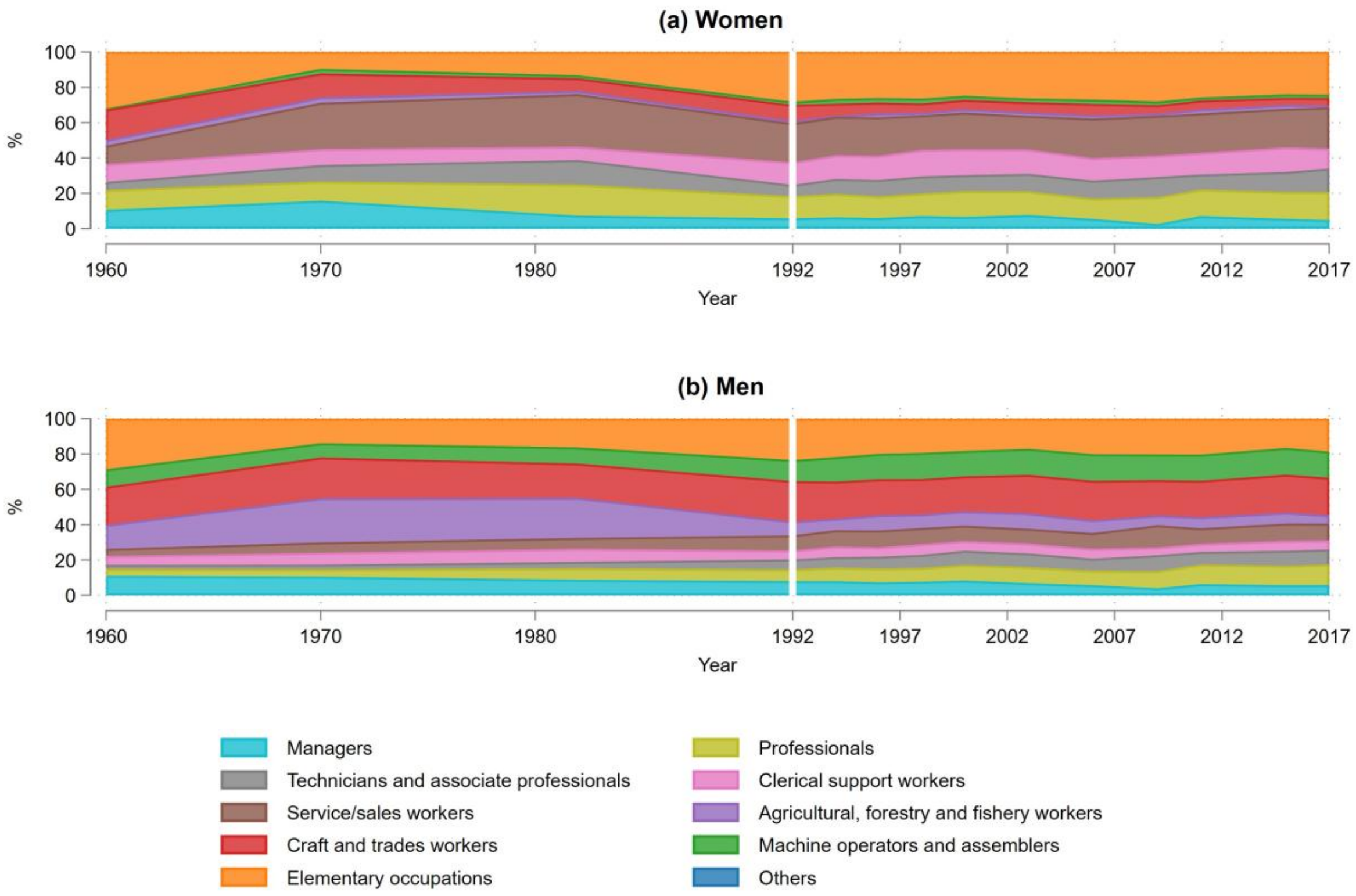

Notes: Data for years 1960, 1970 and 1982 comes from Census data obtained through IPUMS. Data for years 1992, 1994, 1996, 1998, 2000, 2003, 2006, 2009, 2011, 2015 and 2017 comes from the National Socioeconomic Characterization Survey, CASEN.

Supplementary Figure 3

Flowchart of sample selection

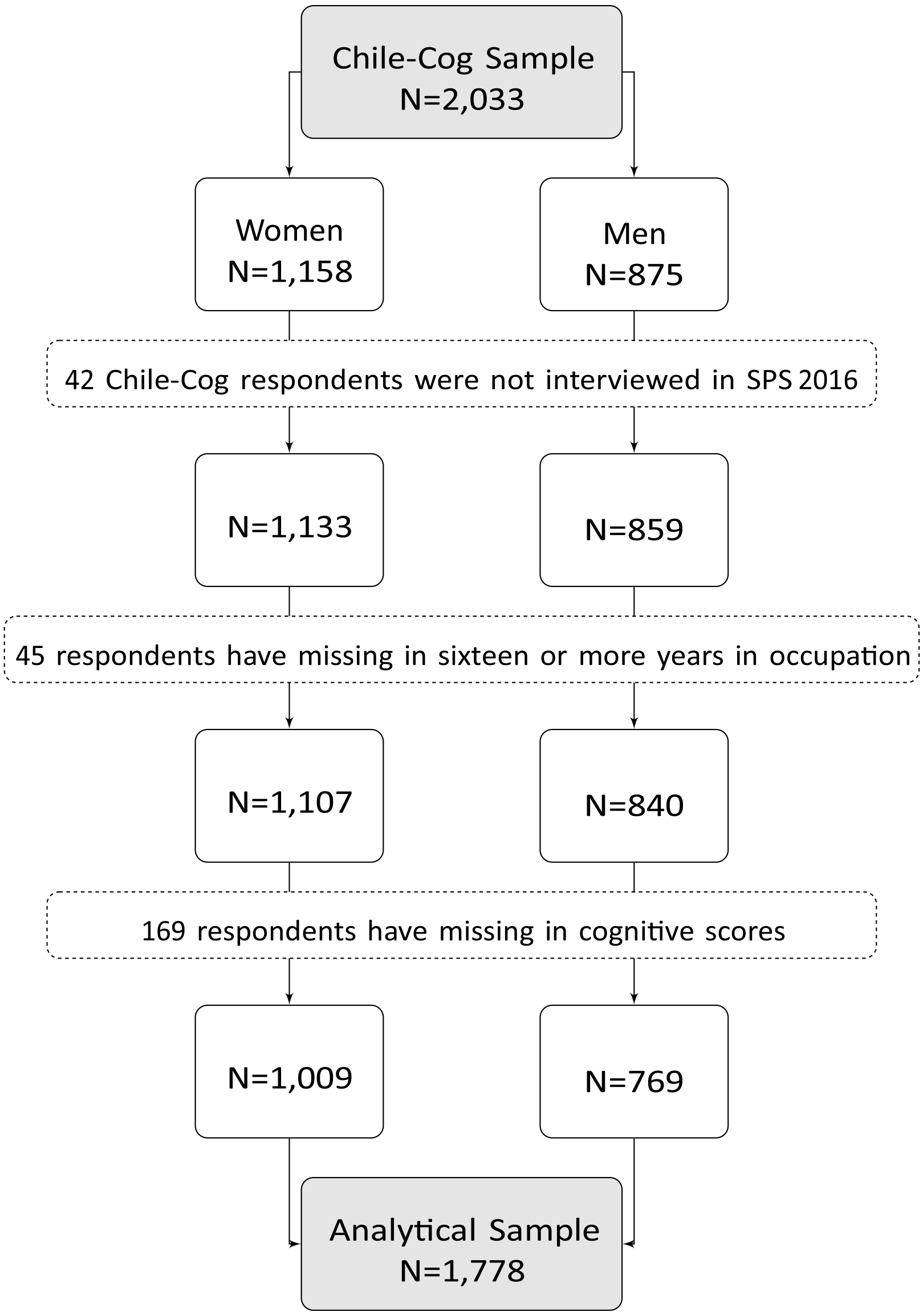

Supplement: Supplementary file 1 [file gbag142_supplementary_data.pdf]
